# Bioengineered 3D Human Trabecular Meshwork Models for Outflow Physiology and Glaucoma Research

**DOI:** 10.3390/bioengineering13030291

**Published:** 2026-02-28

**Authors:** Andrea Valarezo, Pujhitha Ramesh, Rong Du, Rohit Sharma, Evan Davis, Susan T. Sharfstein, John Danias, Yiqin Du, Yubing Xie

**Affiliations:** 1 Department of Nanoscale Science and Engineering, College of Nanotechnology, Science, and Engineering, University at Albany, State University of New York, Albany, NY 12203, USA; avalarezoalban@albany.edu (A.V.); pramesh@albany.edu (P.R.); ssharfstein@albany.edu (S.T.S.); 2 Department of Ophthalmology, Morsani College of Medicine, University of South Florida, Tampa, FL 33612, USA; rongdu@usf.edu (R.D.); rsharma10@usf.edu (R.S.); yiqindu@usf.edu (Y.D.); 3 Departments of Ophthalmology and Cell Biology, SUNY Downstate Health Sciences University, 450 Clarkson Avenue, Brooklyn, NY 11203, USA; evan.davis@downstate.edu (E.D.); john.danias@downstate.edu (J.D.)

**Keywords:** primary open-angle glaucoma, trabecular meshwork, aqueous humor, outflow, 3D culture, in vitro model, hydrogel, scaffold, microfluidic, tissue engineering

## Abstract

Primary open angle glaucoma (POAG) is one of the leading causes of irreversible blindness and is associated with dysfunction of the trabecular meshwork (TM), a three-dimensional (3D) structure that regulates aqueous humor outflow and, consequently, intraocular pressure (IOP). IOP is the only modifiable factor for glaucoma. Outflow facility is the inverse of aqueous humor outflow resistance caused by the presence of the TM and adjacent tissues, and reflects the TM’s central role in IOP control, representing the most physiologically relevant measure of human trabecular meshwork (HTM) function. Therefore, development of ex vivo systems to study outflow facility and IOP regulation is critical for advancing glaucoma research. We present a comprehensive review of bioengineering approaches to generation of 3D HTM models using synthetic, natural, and hybrid hydrogels, micro- and nanofabricated synthetic substrates or porous scaffolds, and microfluidic devices. These 3D HTM systems have been designed to capture key features such as topography, stiffness, and fluid flow in the conventional outflow pathway. In particular, we highlight HTM models that recapitulate IOP regulation and allow measurement of outflow facility, which directly reflect pressure-dependent outflow resistance in dynamic HTM physiology and glaucoma pathophysiology. By integrating these bioengineering approaches with emerging stem cell technologies, this review offers an evidence-based landscape overview and framework for designing next-generation 3D human-relevant TM models for outflow physiological studies and IOP-modulating drug discovery.

## 1. Introduction

Glaucoma is a group of complex, multifactorial optic neuropathies, which leads to progressive optic nerve damage and vision loss [1]. In the United States, over 4 million people have been living with glaucoma, with a prevalence of 2.56% among people 40 years or older [2], of which 50% are undiagnosed and more than 120,000 are blind [3]. Each year, glaucoma costs the United States economy nearly $2.9 billion in direct healthcare costs and indirect costs related to productivity losses [4].

Primary open-angle glaucoma (POAG) is the most common type of glaucoma, representing 85–90% of glaucoma seen in the United States [5]. It remains a prevalent, vision-threatening condition worldwide [6,7], with higher prevalence and severity in certain ethnic groups [8,9,10]. POAG is characterized by the presence of an open, normal-appearing anterior chamber angle between the clear cornea and colored iris, allowing aqueous humor drainage, but a “clogged” trabecular meshwork (TM) reduces aqueous humor outflow and thereby increases intraocular pressure (IOP). The condition is normally painless, and often detected only after partial vision loss, by which time, many of the retinal ganglion cell axons that carry signals from the retina to the brain are irreversibly lost [11,12]. Therefore, if not treated promptly, POAG optic nerve damage can cause irreversible blindness [13].

Elevated IOP is the most significant risk factor for this blinding disease. IOP is also the only modifiable risk factor for glaucoma and a major predictor of long-term disease progression [14,15,16]. Currently, IOP reduction is the only clinically proven intervention to prevent glaucomatous damage and halt disease progression, even when IOP is within the normal range [17,18,19]. Decreased IOP can be achieved using pharmacologic agents, laser trabeculoplasty, and surgery [20,21,22,23,24,25,26,27,28,29,30,31,32]. Evidence from randomized controlled clinical studies shows that effective IOP-lowering therapy significantly reduces glaucoma progression risk at every stage of the disease [33].

**Figure 1:**
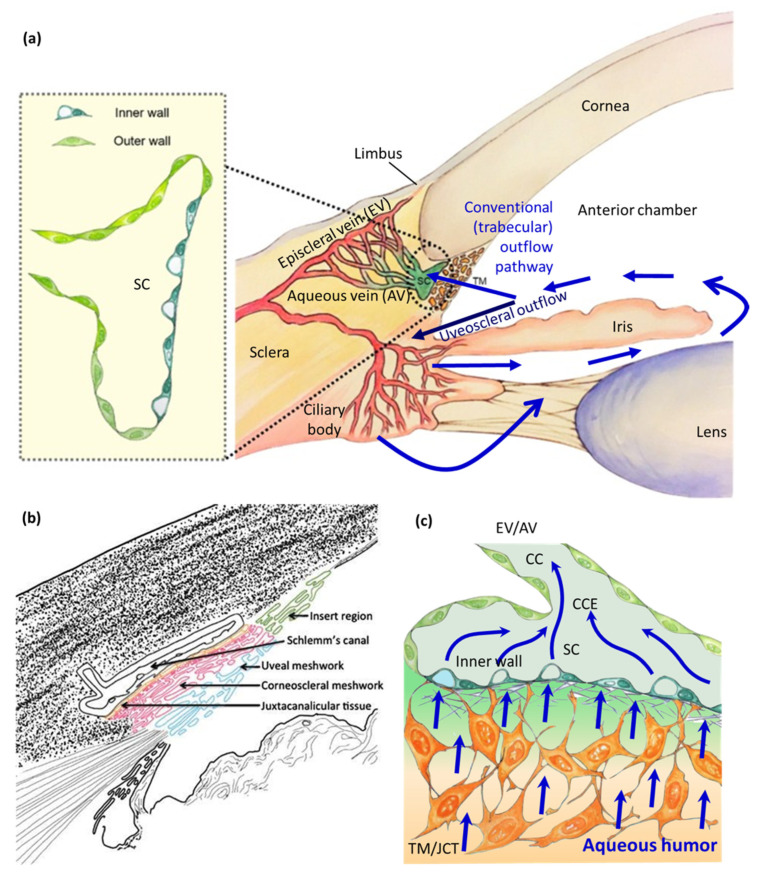
Schematic diagram of outflow pathways and structures in the human trabecular meshwork (HTM). (**a**) Schematic diagram depicting the conventional trabecular outflow pathway and unconventional uveoscleral outflow pathway in the anterior chamber. Insert: An enlarged view of the microanatomy of Schlemm’s canal (SC) lining with inner wall and outer wall SC endothelial cells. Blue arrows indicate the direction of aqueous humor outflow (adapted from Dautriche et al., 2015, J Funct Biomater [54], distributed under the terms of the Creative Commons Attribution license (CC BY 4.0, http://creativecommons.org/licenses/by/4.0/), Copyright © 2015 by the authors, Licensee MDPI, Basel, Switzerland). (**b**) Illustration of the TM structure. Insert region (green, nonfilter region) is between the TM and the corneal endothelium; the TM consists of uveal meshwork (UVM, blue), corneoscleral meshwork (CSM, red), and juxtacanalicular connective tissue (JCT, yellow) (reproduced with permission from Yun et al., 2016, J Ocul Pharmacol Ther, 2016 [37], Copyright © 2016, Mary Ann Liebert, Inc). (**c**) A magnified view of the HTM depicting JCT and the underlining inner wall of SC, extending toward distal outflow regions, including collector channel entrances (CCEs), collector channels (CCs), episcleral veins (EVs), and aqueous veins (AVs). Courtesy of Yangzi Tian.

IOP is determined by the balance between continuous generation of aqueous humor by the ciliary processes (inflow) and aqueous humor elimination (outflow) through the pressure-dependent conventional outflow pathway and pressure-insensitive uveoscleral or unconventional outflow pathway [34], as shown in Figure 1a. In humans, most of the outflow is through the conventional outflow pathway [35,36], which consists of the TM (Figure 1b) [37], including the inner layer uveal meshwork (UVM), middle layer corneoscleral meshwork (CSM), and outer layer juxtacanalicular connective tissue (JCT), as well as the Schlemm’s canal (SC) inner wall endothelium, collector channels (CCs), and aqueous veins (AVs)/drainage vessels [38,39]. The aqueous outflow resistance predominantly stems from the inner wall region of SC, comprising the JCT layer of the TM and its underlining endothelial monolayer of SC cells (Figure 1c), while additional outflow resistance emerges from the distal outflow region, including CC, episcleral veins (EVs), and AVs [40,41,42,43,44,45,46,47].

The TM is an intricate, well-organized, heterogeneous, filter-like 3D connective tissue composed of mechanosensitive TM cells embedded in a fibrillar extracellular matrix (ECM), located in the anterior chamber angle of the eye. The TM and its underlying SC endothelium are the major pathological site of POAG, as well as other open angle glaucomas. TM cells are able to secrete ECM proteins and cytokines [48], and act as the key regulator of IOP [49]. SC cells are specialized, mechanosensitive endothelial cells lining SC, adjacent to the JCT. These SC cells exhibit both vascular and lymphatic characteristics, forming giant vacuoles and transcellular pores that also regulate aqueous humor outflow and IOP.

Stamer and Clark described four TM cell phenotypes: endothelial, fibroblast, smooth muscle, and macrophage-like, based on expression patterns and behaviors [49]. Single-cell RNA sequencing of human TM (HTM) as well as human SC (HSC) tissues revealed two distinct HTM subtypes (myofibroblast- and fibroblast-like cells), and two distinct HSC subtypes (lymphatic- and vascular-like endothelial cells) [50]. Single-cell and single-nucleus RNA sequencing of mouse limbal tissues identified three mouse TM subtypes, including TM1, which is characterized by increased gene expression responsible for ECM structure and metabolism, TM2, which features increased gene expression of secreted ligand signaling that support SC cells, and TM3, which is marked by increased gene expression related to contractile and mitochondrial/metabolic activity [51]. Further transcriptomic profiling of mouse SC cells confirmed lymphatic/vascular characteristics with a predominantly lymphatic phenotype [52]. While comparable TM and SC cell subtypes have been identified across multiple species, including human, cynomolgus and rhesus macaque, pig, and mouse, notable interspecies differences in cell types and gene expression exist [53].

TM cells are a type of specialized cells that rarely divide in the eye under normal conditions [55,56]. Reduction in TM cellularity has been observed in aged and glaucoma patients [57,58,59,60,61,62,63,64]. Loss of TM cellularity in POAG causes naked trabecular beams as well as fusion of trabecular beams, which increases TM stiffness [65]. ECM in the TM is altered in POAG and can affect the physiological state of TM cells and thereby outflow facility. Damage or malfunction of the conventional outflow tissue leads to IOP elevation in humans [66,67,68]. This increased IOP can induce mechanical stress, oxidative stress, mitochondrial dysfunction, and excessive glial cell activation, collectively triggering a gradual (and possibly compartmentalized) degeneration of retinal ganglion cells (RGCs) and their axons over time [69].

Since the original pathology in POAG appears to be in the HTM, development of in vitro models to delineate glaucoma pathophysiology and to support preclinical studies, including IOP-modulating drug screening and testing, is highly desirable. Traditional cell cultures of isolated HTM and HSC cells are useful for studying the biology of these cells and their ECM in normal or glaucomatous states [70,71,72,73,74,75,76,77]. With advances in stem cell technology, pluripotent and multipotent stem cell-derived HTM and HSC cells offer a new avenue for developing well-controlled experimental in vitro model systems. In parallel, bioengineering approaches have attempted to provide environmental cues (e.g., human aqueous humor, nanotopographic surface, substrate rigidity, hydrogel-based scaffolds, micropatterned scaffolds, and microfluidics) for 3D HTM cell culture, mimicking the conventional outflow pathway, in order to understand TM biology and physiology and to test IOP-modulating agents or molecules (Table S1). Notably, 3D HTM culture has demonstrated differences in drug response profiles compared to 2D culture [78,79,80,81,82,83,84], highlighting the importance of using 3D cultures to mimic in vivo HTM behaviors.

In this article, we first review ECM-mimetic hydrogels to recapitulate biochemical and biomechanical properties of the TM microenvironment for HTM culture, including synthetic, natural, and hybrid hydrogels, and decellularized ECM. We then summarize micro-/nanofabricated synthetic substrates that mimic ECM topography, as well as micropatterned porous scaffolds for HTM and HSC cell culture to model the conventional outflow pathway, followed by scaffold-free TM culture platforms. Following that, we discuss the use of stem cells and their integration into 3D HTM and HSC models. Next, we highlight 3D bioengineered systems, such as Artificial Conventional Outflow Systems (ACOSs), which enable perfusion studies and simulated outflow facility measurements, and an ocular fluid outflow on-chip platform, which replicates interstitial fluid drainage through the HTM to SC and enables flow rate measurement under constant pressure. Finally, we provide an outlook on emerging bioengineering approaches to model the conventional outflow pathway, with the goal of advancing our understanding of dynamic HTM physiology and pathophysiology to support therapeutic discovery in glaucoma.

Our objective is to provide a comprehensive, in-depth overview of the field, enabling readers to appreciate how various 3D HTM models replicate key biochemical, biomechanical, and hydrodynamic aspects of the conventional outflow pathway, specifically highlighting the translational importance of physiologically relevant functional readouts, such as outflow facility and pressure-dependent resistance. Research studies related to “3D HTM culture” or “bioengineering HTM” are included in this review; however, recent developments reported only in conference abstracts are not covered due to insufficient details for discussion. This review focuses on the outflow physiology of bioengineered HTM models; readers are referred to review papers by Bikuna-Izagirre et al. for chronological assessment of key technological milestones [85], Ghosh and Herberg for review of ECM biomaterials for modeling TM and SC biology [86], Lamont et al. for biomaterial considerations of TM biomechanical properties and their modulation [87], Buffault et al. for overview of glaucomatous TM models [88], and a book chapter by Torrejon et al. for bioengineering 3D HTM protocols [89].

## 2. Hydrogel-Based HTM Models

Hydrogels provide a 3D or quasi-3D microenvironment that is chemically definable and has adjustable rigidity, capable of modulating cell adhesion, cytoskeletal tension, and ECM mechanics. This category includes both 2D substrates with controlled stiffness and 3D matrices, such as synthetic hydrogels (e.g., polyacrylamide), natural hydrogels (e.g., Matrigel, collagen and its formulations with glycosaminoglycans (GAGs), and gelatin), hybrid hydrogels, peptide hydrogels, and decellularized ECM.

### 2.1. HTM Culture on Synthetic Polyacrylamide Hydrogels

Substrate compliance emerges as a primary regulator of HTM cell biology by regulating cell adhesion, proliferation, morphology, and migrations. Schlunck et al. grew primary HTM cells on 2D polyacrylamide hydrogels (soft vs. stiff) because they are tunable, soft substrates that mimic the mechanical stiffness of native ECM in the TM [90], representing one of the earliest systematic studies to examine the effect of substrate stiffness on HTM cell behaviors. They demonstrated that ECM rigidity modulated HTM cell spreading and focal adhesion size, focal adhesion kinase activation, serum-induced extracellular signal regulated kinase (ERK) phosphorylation, and fibronectin deposition. In the same study, α-smooth muscle actin (αSMA) expression and localization increased on rigid substrates, whereas myocilin and αB-crystallin expression increased on soft substrates [90]. Furthermore, Wood et al. showed that soft (~4 kPa, homeo-mimetic) vs. rigid surfaces (25 kPa, patho-mimetic hydrogel) induced differential cellular responses in terms of cell spreading and morphology, and HTM cells on rigid substrates exhibited greater sensitivity to latrunculin-B (Lat-B) [91], a cytoskeletal actin polymerization inhibitor and experimental IOP-lowering agent. Cell stiffness increased with substrate stiffness and transiently decreased after Lat-B treatment when cultured on a rigid substrate [92]. Increased substrate stiffness significantly promoted expression of glaucoma-associated genes and proteins (e.g., myocilin, secreted protein acidic and rich in cysteine (SPARC)) in HTM cells, and modulated HTM cell expression level in response to Lat-B [93]. Substratum stiffness strongly influences yes-associated protein (YAP)/transcriptional co-activator with PDZ binding motif (TAZ) signaling in HTM cells, with stiff (75 kPa) hydrogels showing elevated YAP/TAZ activity that is rapidly reversed by Lat-B, which alters YAP phosphorylation and localization as well as YAP-regulated, ECM gene expression relevant to glaucoma [94].

Although 2D polyacrylamide hydrogels can simulate the stiffness of normal and glaucomatous HTM, they do not resemble the 3D structure and dynamic outflow of HTM. Therefore, Karami et al. developed a 3D polyacrylamide hydrogel system that mimics the anatomical structure of the conventional outflow pathway by an indirect 3D printing approach [95]. The TM/JCT/SC architecture was reconstructed from serial block-face scanning electron microscopy images using custom segmentation and surface-mesh algorithms, meshing both intertrabecular spaces and open spaces. The geometry of the TM/JCT/SC complex was upscaled 100× due to technical limitations of current 3D printers. The generated stereolithography (STL) file was used to 3D print a mold that replicated the open spaces of the original structure and established a boundary to contain the hydrogel of 315 µm thickness. Polyacrylamide hydrogels embedded with FluoSpheres, serving as displacement markers, were synthesized within the 3D-printed mold to represent normal (1.5 kPa) vs. glaucomatous (21.7 kPa) conditions [95]. These hydrogel scaffolds were coated with type I collagen and seeded with normal HTM cells or HTM cells derived from donors with glaucoma (GTM). The resulting cell–scaffold constructs were perfused at 30 µL/min for 20 h. This platform enabled the visualization and quantification of 3D dynamic traction forces generated by normal and glaucomatous HTM cells within an active fluid–structure interaction environment [95]. Furthermore, it confirmed that matrix stiffness differentially modulated traction forces, cytoskeletal dynamics, and collagen fibril organization in HTM cells under normal and glaucomatous conditions [96].

### 2.2. HTM Culture in Matrigel

Matrigel, a growth factor-rich, tumor-derived ECM, has been used to facilitate 3D culture of various types of cells and organoids [97]. Since it is primarily composed of laminin (~60%) and collagen IV (30%), which is close to the TM ECM composition, Matrigel has been used for 3D HTM culture. Zhang et al. showed that 2D Matrigel (i.e., tissue culture plastic coated with 5% Matrigel) supported the expansion of HTM progenitors more effectively than plastic, fibronectin-, or collagen IV-coated substrates; however, expanded progenitors gradually lost HTM phenotype during serial passages on 2D Matrigel [98]. In contrast, culturing on 3D Matrigel (i.e., hydrogel formulated with 50% Matrigel) enabled cells to regain expression of HTM and stem cell markers [98]. Notably, 3D Matrigel permitted the normal, but not pathological HTM phenotype, even when challenged by a high concentration of transforming growth factor β1 (TGFβ1) [81].

On the other hand, Matrigel-based 3D HTM cultures can be induced to glaucomatous states by treating with pharmacological stressors, such as dexamethasone (DEX) [99], TGFβ2 [99,100], or oxidative stress [101]. Buffault et al. demonstrated that primary HTM cells grown in Matrigel on a cell culture insert exhibited cytoskeletal rearrangements, characterized by the organization of actin into more extensive fibers and a reduction in intercellular space upon TGFβ2 treatment for 48 h, mimicking a diseased phenotype [100]. Subsequent treatment with Y27632 (a selective ROCK inhibitor with IOP-lowering effects) attenuated TGFβ2-induced changes, leading to less extensive actin fibers and widened intercellular spaces; whereas, exposure to latanoprost (a prostaglandin analog) did not alter actin organization or intercellular spacing [100]. Additionally, Bouchemi et al. demonstrated the feasibility of using primary HTM cells cultured in Matrigel on a cell culture insert for toxicological evaluation [99]. They showed that benzalkonium chloride, a preservative commonly used in glaucoma eye drops, triggered secretion of inflammatory cytokines, particularly interleukins (ILs) such as IL-6 and IL-8 [99].

**Figure 2:**
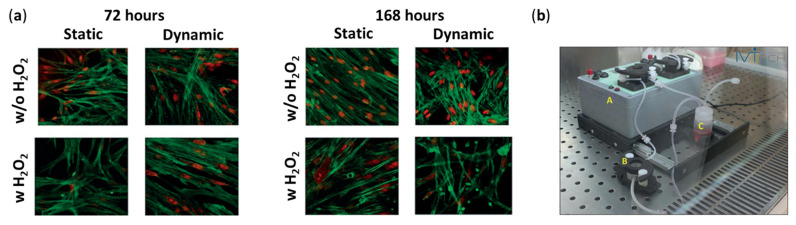
3D-cultured HTM cells in Matrigel. (**a**) Merged confocal images of 3D-cultured HTM cells without any H_2_O_2_ treatment (control) and with 500 µM H_2_O_2_ treatment to mimic chronic stress exposure. H_2_O_2_ was added to respective wells once a day for 2 h, followed by 22 h of recovery under static vs. dynamic perfusion. Samples were assessed at 72 h and 168 h. Red, To-Pro™-stained nuclei. Green, phalloidin-stained F-actin. 60× magnification. (**b**) IVTech perfusion bioreactor setup for dynamic perfusion of the HTM cell–Matrigel construct. Adapted from Saccà et al., 2020, ALTEX [102], under the terms of the Creative Commons Attribution 4.0 International License (http://creativecommons.org/licenses/by/4.0/), Copyright © 2020 by the authors.

In oxidative stress-induced glaucoma models, H_2_O_2_ treatment of 3D-cultured HTM in Matrigel has shown to induce a glaucomatous phenotype, with the potential to mimic key stress-related pathological features in glaucoma, such as mitochondrial dysfunction and ECM dysregulation. Vernazza et al. embedded HTM cells in Matrigel in culture wares and demonstrated increased sensitivity to reactive oxygen species (ROS)-induced damage, compared to conventional 2D cultures [84]. Saccà et al. further incorporated dynamic perfusion into 3D cultures of HTM cells in Matrigel (Figure 2), in which the culture medium was circulated with basal perfusion at a high, non-physiological flow rate (70 µL/min―the chosen flow rate to overcome diffusional limitations while avoiding Matrigel^®^ degradation over time) [102]. Dynamic perfusion preserved HTM function over time and allowed inflammatory pathway activation under chronic oxidative stress induced by H_2_O_2_ treatment, in comparison to static culture [102]. Furthermore, they demonstrated the feasibility of evaluating the short-term anti-inflammatory and antioxidant effects of iTRAB^®^, a patented formulation of polyphenols and fatty acids/stress response inhibitor, on mitigating ROS-induced HTM cell damage [103], as well as the protective effect of citicoline (a neuroprotective compound) against oxidative stress on HTM cells [104]. These foundational studies demonstrate that Matrigel facilitates the development of 3D HTM disease models for toxicological evaluation and antioxidant assessment of anti-glaucoma agents.

### 2.3. HTM Culture in Collagen-Based Hydrogels

As one of the major components of ECM [105], collagen-based hydrogels have been widely adopted in HTM cell culture, providing an alternative to mouse tumor-derived and ill-defined Matrigel. 3D culture of HTM cells in collagen matrices has demonstrated enhanced physiological relevance, mimicking native ECM environments more effectively than 2D models. These systems promote cellular morphology, gene expression, and mechanical responses that closely resemble in vivo HTM tissue, offering improved models for studying glaucoma and aqueous humor outflow physiology.

**Figure 3:**
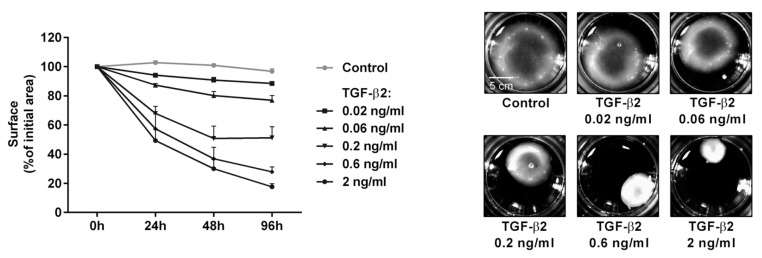
3D HTM cell-populated collagen gel model exhibited dose-dependent contraction in response to TGFβ2 (0–2 ng/mL). (**Left panel**) Quantification of contraction. (**Right panel**) Illustrations of the 3D HTM-populated collagen at 96 h (reproduced from Kalouche et al., 2016, Invest Ophthalmol Vis Sci [106], under the terms of the Creative Commons Attribution–NonCommercial–NoDerivatives (CC-BY-NC-ND) 4.0 International License (https://creativecommons.org/licenses/by-nc-nd/4.0/)).

Kalouche et al. developed a 3D HTM-populated collagen gel model using type I collagen, which demonstrated distinct effects of prostaglandin F prostanoid receptor (FP) and prostaglandin E_2_ receptor 2 (EP2) activation on myofibroblast transition [106]. In response to latanoprost (a prostaglandin F2α analogue and the first FP agonist), the 3D HTM model exhibited reduced collagen accumulation but an enhanced contractile phenotype (Figure 3) [106]. The 3D HTM model was also responsive to TGFβ2 in a dose-dependent manner, and additional stimulation with butaprost (an EP2 agonist) attenuated HTM contraction and collagen deposition induced by TGFβ2 [106].

**Figure 4:**
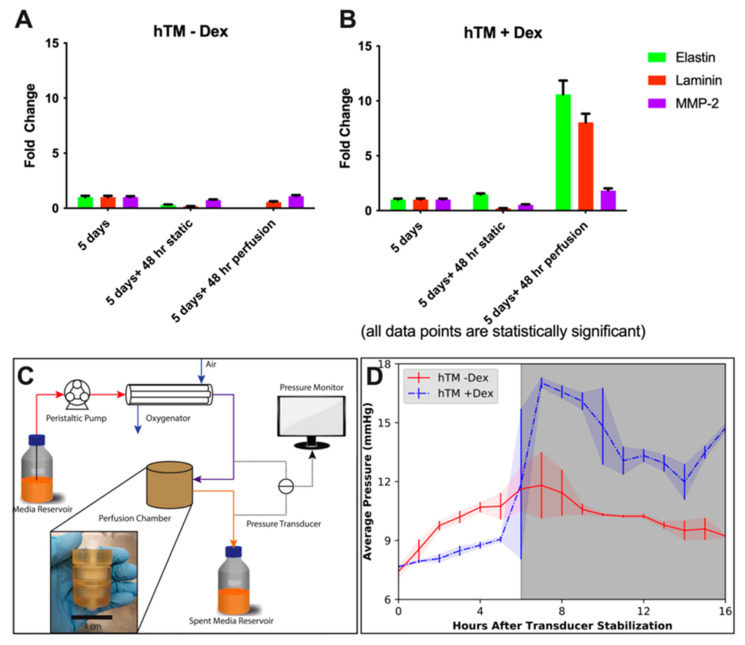
HTM cells grown on collagen–CS hydrogels for 5 days were responsive to 2-day DEX treatment. (**A**,**B**) qPCR analysis of elastin, laminin, and MMP2 expression in the absence of DEX (**A**) and presence of DEX (**B**). (**C**) Schematic of the perfusion platform. (**D**) DEX treatment increased the pressure across the cell–scaffold construct during perfusion over time. Reprinted with permission from Adhikari et al., 2022, ACS Biomater Sci Eng [109], Copyright © 2022 American Chemical Society.

Alternatively, Osmond et al. fabricated type I collagen and collagen–chondroitin sulfate (CS) scaffolds with uniaxially aligned pores by unidirectional freezing and lyophilization [107]. This work is technologically innovative in the development of biomimetic 3D HTM scaffolds. Both collagen and collagen–CS scaffolds supported porcine TM cells to grow into 3D cell–scaffold constructs for two weeks [107]. Furthermore, they evaluated the role of biophysical cues in regulating HTM cell behaviors by fabricating collagen scaffolds with non-aligned vs. aligned and large vs. small pores [108]. Collagen scaffolds with non-aligned large pores supported the best HTM cell growth and gene expression of fibronectin [108]. Further modification of these collagen scaffolds with GAGs, including CS, hyaluronic acid (HA), and CS + HA, demonstrated that HTM cell growth and expression of fibronectin were affected by GAG composition, which was also influenced by the pore architecture of GAG-modified collagen scaffolds [108]. Furthermore, these GAG modifications, in particular, collagen–CS, altered the expression of other ECM proteins, such as elastin and laminin, as well as matrix metalloproteinase 2 (MMP-2) in HTM, which are significantly upregulated and associated with pressure elevation in response to perfusion with DEX (at a single, high, non-physiological flow rate of 61.09 μL/min) (Figure 4) [109].

**Figure 5:**
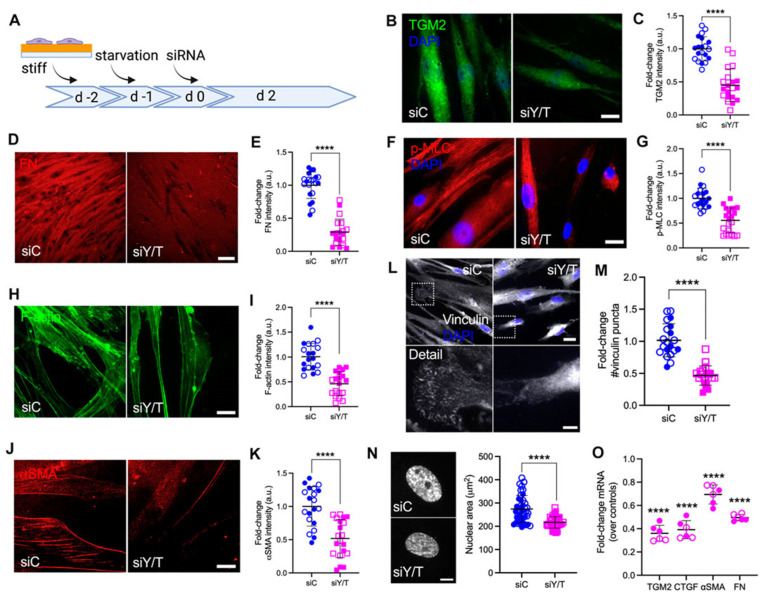
Primary HTM cells grown on stiffened collagen–HA hydrogels treated with siRNA knockdown to deplete YAP and TAZ demonstrated YAP/TAZ regulation of focal adhesion formation, ECM remodeling, and cell contractile properties. (**A**) Schematics of treatment. (**B**,**D**,**F**,**H**,**J**,**L**) Representative fluorescence images and (**C**,**E**,**G**,**I**,**K**,**M**) quantification of transglutaminase 2 (TGM2), fibronectin (FN), phosphorylated myosin light chain (p-MLC), F-actin, αSMA, and vinculin expression, (**N**) nuclear size analysis, and (**O**) qRT-PCR analysis of TGM2, connective tissue growth factor (CTGF), αSMA, and FN in HTM cells on stiff hydrogels subjected to siRNA Control (siC) or YAP and TAZ siRNA (siY/T). Scale bars: 20 μm (**B**,**F**,**H**,**J**), 100 μm (**D**), and 20 μm (**L**, **top panel**) and 5 μm (**L**, **bottom panel**). ****, *p* < 0.0001. Reproduced from Li et al., 2022, Front Cell Dev Biol [113] under the terms of the Creative Commons Attribution (CC BY) License (http://creativecommons.org/licenses/by/4.0/), Copyright © 2022 Li, Raghunathan, Stamer, Ganapathy, and Herberg.

Furthermore, collagen–HA hydrogels have been synthesized by UV-crosslinking a mixture of primary HTM cells with methacrylated collagen, thiolated HA (containing photoinitiator), and elastin-like polypeptide [110]. Li et al. developed 3D normal and glaucomatous HTM models by culturing primary HTM cells from donors without or with glaucoma on collagen–HA hydrogels, respectively [111]. This work is methodologically important, contributing to a valuable hydrogel-based platform for advanced glaucoma disease modeling. These models were responsive to DEX, demonstrating increased contractility, expression and rearrangement of F-actin and α-SMA, fibronectin deposition, and stiffening, all of which were mitigated by ROCK inhibitor Y27632 treatment [111]. Additionally, Bague et al. demonstrated the effect of netarsudil-family ROCK inhibitors on reversing TGFβ2-induced pathologic HTM cell contractility and actin remodeling [112]. To further account for biomechanical changes observed in glaucoma where GTM is ~1.5−5-fold stiffer than normal HTM, Li et al. modulated their collagen–HA hydrogels using riboflavin-mediated secondary UV-crosslinking, to achieve ∼2-fold stiffening [113]. Stiffened collagen–HA hydrogels seeded with HTM or GTM cells demonstrated larger-sized nuclei, enhanced F-actin, and actomyosin cytoskeletal rearrangement correlating with YAP/TAZ nuclear localization in cells, along with increased α-SMA expression and fibronectin deposition compared to soft collagen–HA [113]. Furthermore, TGFβ2-increased nuclear YAP/TAZ in both HTM and GTM cells was prevented by inhibiting ERK and ROCK signaling pathways. YAP/TAZ inhibition with verteporfin significantly reduced TGFβ2-induced contractility and stiffening in both HTM and GTM cells within soft 3D hydrogel environments [113]. Moreover, combined siRNA knockdown of YAP and TAZ demonstrated that YAP/TAZ mediate focal adhesion formation, ECM remodeling, and cell contractile properties of HTM grown on stiff collagen–HA hydrogels (Figure 5) [113].

Karimi et al. also evaluated the role of biomechanics in segmental flow of normal or glaucomatous HTM in collagen hydrogels since aqueous humor outflow through the TM is segmental, with distinct high-flow and low-flow regions [96]. High-flow HTM/JCT or GTM cells were embedded in type I collagen hydrogels with tunable stiffness (4.7 and 27.7 kPa) through polymerization at 37 °C; they showed that matrix stiffness differentially modulated traction forces, cytoskeletal dynamics, and collagen fibril organization in HTM and GTM cells [96]. The role of cytoskeletal filaments in regulating TM cell biomechanics was delineated using 3D traction force microscopy, coupled with fibril strain mapping. Normal high-flow HTM/JCT cells in collagen hydrogels (elastic modulus 4.7 kPa) were treated with Lat-B (actin depolymerization), nocodazole (microtubule depolymerization), or withaferin A (intermediate filament disassembly) for selective inhibition of specific cytoskeletal changes, revealing that the actin generates and microtubules sustain ~80% of HTM traction, whereas loss of intermediate filament has a neutral effect [114]. This study highlights their synergistic role in maintaining TM biomechanics and suggests that reducing traction forces by 10 kPa (~80%) at the cell–matrix interface may be sufficient for HTM/JCT cells to modify segmental outflow resistance [114].

Lamont et al. developed a collagen-based TM model highlighting the role of scaffold architecture in mimicking the JCT region [115]. HTM cells were grown on conventional type I collagen hydrogels vs. plastically compressed collagen hydrogels (with high stiffness and JCT-like aligned collagen architecture) in the absence or presence of a controlled and spatially released TGFβ2 [115]. Plastically compressed collagen hydrogels provided biophysical cues that induced JCT-like cellular characteristics, including increased elastin expression and sustained αB-crystallin protein expression, cytoskeleton remodeling, and increased gene expression of mesenchymal and JCT-specific markers, in comparison to conventional type I collagen hydrogels [115]. The JCT-like bioengineered tissue responded to local TGFβ2 exposure and demonstrated a pathological mesenchymal phenotype [115].

Gelatin, a denatured form of collagen, has also been explored for bioengineering HTM models due to its ease of sourcing and handling. Particularly, gelatin methacryloyl (GelMA), a photo-crosslinkable form of gelatin offering tunable mechanical properties and superior stability at physiological temperatures, has been explored for 3D HTM culture. Li et al. demonstrated that soft GelMA hydrogels support the physiologically relevant TM phenotype in GTM3L cells with lower crosslinked actin network (CLAN) formation compared to cells grown on a stiff glass coverslip [77]. Adhikari et al. biochemically modified GelMA with GAGs and showed that the CS-modified GelMA hydrogel increased expression of fibronectin and α-SMA while the HA-modified hydrogel attenuated fibrotic features [116]. Furthermore, they developed a simplified and reproducible 3D-printed HTM model using GelMA bioinks with a well-defined grid and pore structure, which responded to DEX treatment by increased expression of fibronectin [117]. Perfusion was performed at a high, non-physiological flow rate of 61 µL/min for 48 h for dynamic culture to overcome nutrient and oxygen diffusion limitations, which significantly increased cell proliferation compared to static culture [117]. The 3D-printed system provides guidance towards designing and fabricating well-defined, structured hydrogel scaffolds for HTM outflow studies, offering a methodologically important and tunable 3D-printed GelMA platform for perfusion-based HTM and glaucoma research.

### 2.4. HTM Culture in Hybrid Mechanoelectric Transducing Hydrogels

To develop functional hydrogels for HTM culture, Wang et al. synthesized acrylamide (AM)/GelMA/HA hydrogels using both synthetic and natural hydrogel components, taking advantage of AM’s added mechanical strength and tunable stiffness, GelMA’s adhesion property, and HA’s ECM mimicry and enhanced hydration [118]. Mechanoelectric transducing hydrogels were further developed by incorporating MXene nanosheets. MXene is a growing family of 2D materials (transition metal carbides, nitrides, and carbonitrides) with a layered structure and tunable electronic/optical/mechanical properties. MXene provided electrical conductivity and served as the mechanoelectric transducer in the 3D HTM model. The glaucomatous phenotype was induced in the 3D HTM model by TGFβ2, oxidative stress (H_2_O_2_ treatment), or high pressure. The high-pressure model was further used to test the potential therapeutic potential of piezoresponsive nanomaterials (such as MXene) for restoring HTM-mediated regulation of IOP homeostasis. In this system, in vitro cyclic pressure―simulating aqueous humor outflow and HTM contraction in vivo―induced piezoelectric stimulation of HTM cells, generating electrical signals that in turn activate large-conductance calcium-activated potassium channels. Activation of these channels regulates cell volume and contractility, ultimately reducing IOP. Furthermore, MXene’s ability to lower IOP was confirmed in a rabbit transient ocular hypertension model in vivo [118]. This technologically novel work represents an emerging therapeutic direction in glaucoma research.

### 2.5. HTM Culture in Self-Assembed Peptide Hydrogels

The peptide-based hydrogel is another example of emerging functional hydrogels, which offers advantages over protein-based biomaterials for bioengineering 3D HTM models, due to controllable synthesis, precise functionalization, enhanced stability, and lower immunogenicity. Waduthantri et al. synthesized a biocompatible MAX8B peptide hydrogel for HTM culture [119]. MAX8B refers to a blend of two peptides―MAX8 (synthetic β-hairpin peptides) and MAX8 GRGD (a modified version that includes the GRGD (glycine–arginine–glycine–aspartic acid) peptide motif), which can fold and self-assemble into an injectable, well-defined, shear-thinning, nanofibrillar hydrogel. The MAX8B hydrogel supported HTM cell viability and proliferation [119]. The HTM cell–hydrogel construct was perfused with the vehicle control or DEX at a single physiological flow rate of 3 µL/min for 7 days, and pressure across the cell–hydrogel construct was recorded over time using a differential pressure transducer in a perfusion system, showing DEX-induced pressure elevation over time [119].

### 2.6. HTM Culture on Decellularized Extracellular Matrices

Decellularized extracellular matrices offer an excellent platform for bioengineering 3D models due to their native ECM architecture, biochemical composition, and biomechanical properties. Crouch et al. optimized a protocol to decellularize HTM tissues, involving treatment with NH_4_OH (2% *v*/*v*, 2 h), washing with Triton X-100 (1% *v*/*v*, 16 h), immersion in DNase (1% *w*/*v*, 30 min), and three washes in TRIS-buffered saline (50 mM, 30 min each), which gives rise to fully decellularized matrices with limited structural damage [120]. However, native tissues, including TM tissues, are in limited supply and insufficient to meet the demands of high-throughput preclinical research. To overcome this supply limitation and to fully recapitulate HTM ECM, Raghunathan et al. generated cell-derived matrices by culturing HTM cells on glass coverslips in the absence or presence of DEX or TGFβ for four weeks, followed by decellularization with 20 mM NH_4_OH and 0.05% Triton X-100 [121,122,123]. They further demonstrated that GTM-derived matrices induce stiffening and glaucomatous-like gene and protein expression in normal HTM cells, while also triggering endoplasmic reticulum (ER) stress [124], highlighting the critical role of glaucomatous ECM in modulating TM cell behavior. Their results are consistent with findings by Kasetti et al., who reported that glaucomatous ECM (derived from DEX-treated HTM cells) induced ER stress in non-glaucomatous HTM cells [75].

### 2.7. Limitations of Hydrogel-Based HTM Models for Perfusion Studies

Hydrogel-based HTM models can recapitulate ECM biochemistry and biomechanics, providing an excellent in vitro system for studying HTM biology, ECM remodeling, biomechanics of HTM and its ECM, and drug response. However, these hydrogel-based HTM systems have limitations for pressure-dependent outflow studies.

For example, the Matrigel-based HTM model was integrated with a perfusion bioreactor, but the perfusion served only as a dynamic culture condition, rather than an outflow function assessment platform, and was applied at a high, non-physiological flow rate (70 µL/min) to overcome nutrient and oxygen diffusion limits [102]. Matrigel is extremely soft and fragile, and therefore not stable for sustained flow to measure outflow facility. Similarly, the 3D-printed GelMA-based HTM model was perfused at a high, non-physiological flow rate (61 µL/min) to overcome diffusion limits as well [117]. However, the large pore size (0.5–2 mm) and non-uniform cell distribution in 3D-printed constructs make them impractical for measuring outflow facility at physiological flow rates.

The collagen–CS hydrogel-based model utilized constant flow perfusion at a high, non-physiological flow rate (61.09 µL/min) [109]. Although the peptide hydrogel-based HTM model permitted perfusion at a physiological flow rate (e.g., 3 µL/min) [119], both hydrogel-based systems generated only transmembrane pressure vs. time graphs in response to drug treatment. Because measured pressure did not reach a steady state, calculating outflow facility was not feasible.

These limitations likely arise from the fact that hydrogels are too soft and deform under pressure, swell or collapse under flow, exhibit uncontrolled hydraulic resistance due to variable pore size and time-dependent changes in permeability, and lack long-term mechanical stability because of fatigue under repeated loading. The design of strong, tough hydrogels [125] combined with 3D bioprinting [117] has the potential to overcome this limitation, achieving hydrogels with ECM-mimetic viscoelasticity while maintaining strong mechanical stability, minimum swelling, and well-controlled pore size and architecture.

## 3. Micro- and Nanofabricated Substrate-Based HTM Models

While micro- and nanofabricated nonporous substrates (Section 3.1) can be used to mimic TM basement membrane topography for modulating HTM cell behavior, micro- and nanofabricated porous scaffolds have been designed and fabricated to replicate the JCT, CSM, and UVM regions of the multilayered architecture of HTM. Examples include: (1) non-biodegradable, micropatterned porous SU-8 scaffolds fabricated by photolithography to mimic the JCT layer for HTM and HSC culture (Section 3.2), and (2) biodegradable, porous poly-ε-caprolactone (PCL) scaffolds fabricated by the sacrificial layer technique, electrospinning, cryoelectrospinning, and melt electrowriting (MEW) to construct the full HTM structure (Table 1) (Section 3.3). These porous scaffolds facilitate the development of an interfacial tissue that can be modularly built into perfusion systems to simulate fluid flow that mimics aqueous humor drainage, thereby allowing simulated outflow measurements or IOP modulating studies.

### 3.1. Micro- and Nanopatterned Nonporous Polyurethane Surfaces for HTM Culture

Russell et al. pioneered the work to examine how nanotopography influences HTM cell behavior. Using soft lithography, they fabricated nanopatterned polyurethane surfaces with an anisotropic array of ridges and grooves that mimicked fibers present in the basement membrane [126]. This work revealed that HTM cell alignment and elongation oriented with the grooves on the patterned surface and expression of glaucoma-associated markers, such as myocilin and versican, was enhanced compared to the control (flat polyurethane surface) [126]. Although nanopatterned surfaces influenced these cellular behaviors, they did not alter oxidative stress-induced IL-6 mRNA stability [127]. Additionally, Kim et al. showed that micropatterned aligned structures enhanced cell alignment while reducing myocilin expression compared to other topographic structures [128]. These studies underscore the importance of surface topography in TM biology.

### 3.2. Micropatterned Porous SU-8 Scaffolds for HTM and HSC Culture to Develop Artificial Conventional Outflow Systems (ACOSs)

To mimic the filter-like, conventional outflow pathway, Torrejon et al. established a foundational and highly influential framework [129], designing and fabricating micropatterned, porous SU-8 scaffolds using photolithography, to mimic the JCT region interfacing TM and SC, culturing primary or stem cell-derived HTM and/or HSC cells, and recapitulating outflow physiology [129,130,131,132]. They established ACOSs that permit monitoring of cell phenotype and, in particular, the measurement of simulated outflow facility [89] (see Section 6.2 for more details).

**Figure 6:**
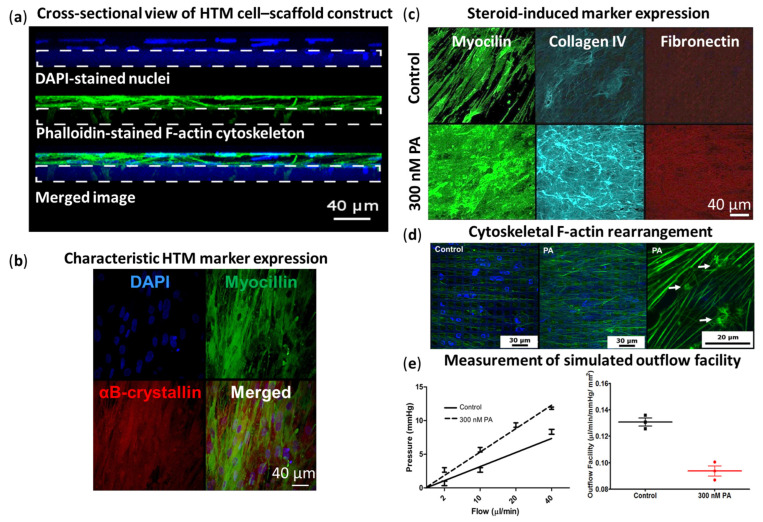
Characterization and validation of the Artificial Conventional Outflow System (ACOS). (**a**) Confocal images of the cross-sectional view of the HTM cell–scaffold constructs. (**Top**) DAPI-stained nuclei in blue. (**Middle**) Phalloidin-stained F-actin cytoskeleton in green. (**Bottom**) Merged image. The dashed box denotes the SU-8 scaffolds with autofluorescence in the blue channel. (**b**) Confocal images show expression of characteristic HTM markers of myocilin (in green) and αB-crystallin (in red) in the HTM cell–scaffold construct, co-stained with DAPI to show the nuclei of the total cell population (in blue). (**c**,**d**) Confocal images of HTM cell–scaffold constructs showed increased expression of myocilin, collagen IV, and fibronectin after being perfused with 300 nM prednisolone acetate (PA) for 9 days (**c**), along with steroid-induced cytoskeletal rearrangement phalloidin-stained F-actin and formation of CLANs (white arrows) (**d**). (**e**) The responsiveness of the ACOS to steroid treatment was functionally confirmed by reduced outflow facility after being perfused with 300 nM PA for 9 days compared to perfusion with the vehicle control (**right**), which is calculated from the graph of pressure vs. flow rate (**left**). (**a**,**b**) Reproduced with permission from Torrejon et al., 2013, Biotechnol Bioeng [129], Copyright © 2013 Wiley Periodicals, Inc. (**c**–**e**) Reproduced with permission from Torrejon et al., 2016, Biotechnol Bioeng [133], Copyright © 2016 Wiley Periodicals, Inc.

SU-8 is a biocompatible, lithography-definable, negative photoresist, widely used in micro- and nanofabrication. Torrejon et al. microfabricated well-defined, porous SU-8 scaffolds with 12 µm pore size and 7 µm beam width (the distance between two neighboring pores) for 3D HTM models [129]. They demonstrated that 12 µm gelatin-coated SU-8 scaffolds supported the best HTM cell growth compared to 7 µm and 15 µm pore sizes, producing a complex, confluent, multilayered HTM structure (Figure 6a), embedded in ECM and reminiscent of the JCT [129]. They further characterized the 3D HTM model by confirming expression of HTM markers (e.g., myocilin, αB-crystallin) (Figure 6b) and ECM proteins (e.g., fibronectin, collagen IV). Furthermore, they validated the 3D HTM model as steroid-responsive and developed a steroid-induced glaucomatous model by treating 3D-cultured HTM cells with prednisolone acetate (PA), which induced increased myocilin expression, ECM protein deposition (Figure 6c), and CLAN formation (Figure 6d), and reduced simulated outflow facility (Figure 6e) [129,133], mirroring pathophysiology observed in vivo.

This 3D HTM model also responded to other IOP-modulating agents, including IOP-lowering Lat-B and ROCK inhibitor Y27632 and IOP-elevating TGFβ2 and steroid DEX, demonstrating the expected characteristics [129,131,133,134]. For example, in response to Lat-B, an IOP-lowering agent, HTM cells cultured on SU-8 scaffolds exhibited morphological changes from an elongated spindle-like morphology to a square-like shape, accompanied by disruption of the underlying secreted ECM and reorganization of the cytoskeleton from elongated actin fibers to punctate actin bundles while reducing the outflow resistance [129]. In addition, IOP-lowering agents, such as Lat-B and Y27632 can reduce the resistance in PA-induced glaucomatous HTM [133] and restore outflow facility to the normal control level in TGFβ2-induced glaucomatous HTM [134], respectively.

Dautriche et al., on the other hand, demonstrated the feasibility of culturing HSC cells on micropatterned, porous SU-8 scaffolds, replicating the inner wall of the SC [130]. SU-8 scaffolds coated with Extracel, a hyaluronic acid- and gelatin-based hydrogel, better supported HSC cell growth than those coated with gelatin alone, exhibiting robust expression of HSC-characteristic markers, VE-cadherin and CD31, as well as Fibulin-2, an ECM protein enriched in the inner wall of the SC [130]. Furthermore, these HSC cell–scaffold constructs were responsive to TGFβ2 treatment, exhibiting increased F-actin stress fiber formation, decreased expression of HSC markers (VE-cadherin and CD31), and increased expression of myofibroblast marker (αSMA) and ECM proteins (fibronectin, collagen I and collagen IV) [130].

Tian et al. further advanced 3D HTM models by developing a 3D HTM/HSC co-culture model on gelatin-coated SU-8 scaffolds, to more closely mimic the conventional outflow pathway. They mimicked the TM–inner SC wall interface by culturing primary HTM cells on one side of the scaffold and primary HSC cells on the reverse side of the HTM cell–scaffold construct [132]. This model was characterized and validated by response to DEX treatment as expected, e.g., decreased simulated outflow facility, increased expression of ECM proteins such as collagen IV and fibronectin, and altered cytoskeletal F-actin expression after treatment with 100 nM DEX for up to 7 days [132].

Currently, the ACOS is, to the best of our knowledge, the only in vitro 3D culture system that enables monitoring of both cell phenotype and simulated outflow facility. However, as far as the mechanical properties are concerned, the elastic modulus of SU-8 (typically 2–5 GPa) is substantially higher than that of HTM (~4 kPa), limiting full replication of the tissue microenvironment. Further development of micropatterned porous scaffolds to better match mechanical properties of HTM ECM will enable the ACOS to more faithfully replicate native HTM.

### 3.3. Porous PCL Scaffolds for HTM Culture

**Figure 7:**
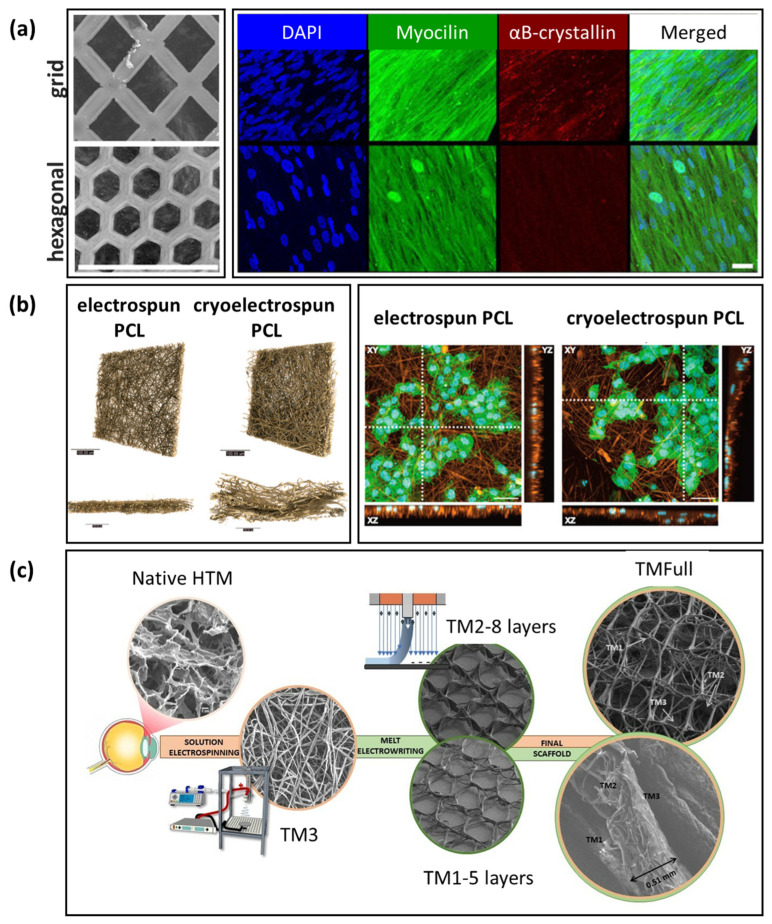
Porous PCL scaffolds for HTM culture. (**a**) Micropatterned porous PCL scaffolds with grid (top) or hexagonal patterns (bottom). Left panels, scanning electron microscopy (SEM). Scale bar = 40 µm. Right panels, confocal images of HTM cells cultured on PCL scaffolds for 14 days, expressing HTM markers of myocilin and αB-crystallin. Scale bar = 30 µm (adapted from Beardslee et al., 2023, Bioengineering [135], under the terms and conditions of the Creative Commons Attribution (CC BY) License (https://creativecommons.org/licenses/by/4.0/), Copyright © 2023 by the authors, Licensee MDPI, Basel, Switzerland). (**b**) Electrospun and cryoelectrospun PCL scaffolds. Left panel, X-ray computed tomography images presented in front-on (**top**) and cross-sectional orientations (**bottom**), showing cryoelectrospun PCL increased pore size and thickness compared to electrospun PCL scaffolds. Scale bars = 100 µm. Right panel, confocal z-stacked images of NTM5 cells after 7 days in culture on electrospun PCL and cryoelectrospun PCL scaffolds. Immunocytochemistry staining of cell nucleus (DAPI, blue), cytoskeleton (phalloidin-488, green), and fibers (rhodamine, orange). Images shown: XY z-stack (20× objective, scale bar = 50 µm), XZ, and YZ side view. White dashed line on XY z-stack represents position of XZ (horizontal) and YZ (vertical) images (adapted from Crouch et al., 2023, J Funct Biomater [137], under the terms and conditions of the Creative Commons Attribution (CC BY) license (https://creativecommons.org/licenses/by/4.0/), Copyright © 2023 by the authors, Licensee MDPI, Basel, Switzerland). (**c**) Electrospinning in combination with melt electrowriting (MEW) to generate an electrospun JCT-like layer (TM3) and MEW CTM-like (TM2) and UVM-like (TM1) stacks, which were assembled into the full HTM structure (TMFull) (reproduced from Bikuna-Izagirre et al., 2024, Polymer [141], under the terms and conditions of the Creative Commons Attribution (CC BY) license (https://creativecommons.org/licenses/by/4.0/), Copyright © 2024 by the authors, Licensee MDPI, Basel, Switzerland).

PCL is a biodegradable and mechanically softer biomaterial than non-biodegradable, stiff SU-8, and it can be micro- or nanofabricated to mimic JCT or JCT/CSM/UVM ECM architecture of the HTM (Table 1), with well-defined features suitable for perfusion studies. For example, Beardslee et al. fabricated ultrathin, micropatterned PCL scaffolds using a sacrificial layer technique (Figure 7a), which supported HTM cell growth, cytoskeletal organization, and expression of HTM markers and ECM proteins (fibronectin, collagen IV, and laminin) [135].

Izagirre et al. demonstrated the feasibility of using electrospun nanofibrous PCL scaffolds to mimic the JCT. The HTM cell–scaffold construct exhibited HTM-like features and response to IOP-elevating DEX and IOP-lowering netarsudil as expected, providing a JCT model for investigation of outflow physiology and screening IOP-modulating pharmacological agents [136]. Crouch et al. further demonstrated that cryoelectrospinning generated cryoelectrospun PCL scaffolds with increased porosity, pore size, and thickness, which improved penetration of NTM5 cells (a transformed human normal trabecular meshwork 5 cell line), compared to conventional electrospun PCL scaffolds (Figure 7b) [137].

Using a MEW technique that allows the generation of graded porosity and multilayer stacks, Włodarczyk-Biegun et al. designed and printed porous PCL scaffolds up to 88 layers, with tunable properties, to mimic the multilayer structure of native HTM, pioneering a technologically innovative and methodologically important fabrication strategy that enhances the structural fidelity of engineered HTM scaffolds [138]. These PCL scaffolds supported primary HTM cell growth into confluence and expression of HTM marker αB-crystallin [138]. It is noteworthy that although HTM cells grown on PCL scaffolds remained metabolically active, their viability decreased from 70–90% on day 1 to 50–70% by day 14 [138]. This decrease in viability may be attributed to the thick, stacked layers and small pores in the JCT-mimicking regions, which restrict diffusion and lead to local hypoxia, nutrient depletion, and waste accumulation, particularly by day 14. This observation underscores the critical challenge of limited oxygen and nutrient diffusion in dense scaffold architectures used for chronic glaucoma modeling. On the one hand, dynamic perfusion or rotary culture can enhance oxygen and nutrient transport and improve waste removal, thereby supporting long-term cell viability. On the other hand, bioengineering strategies, such as designing thinner scaffold regions, integrating microchannels to increase permeability, or employing composite scaffolds with high porosity, may also help address this challenge.

**Table 1:** Properties of porous SU-8 and PCL scaffolds mimicking a multilayer HTM structure (first row).

HTM Structure	Fabrication Method	Thickness	Fiber Diameter	Pore Size/ Porosity	Mechanical Properties	Refs.
JCT	Native	2–20 μm	5–12 μm	4–7 µm		[138,139,140]
CSM		40–60 µm	Lamellar	30 µm		
UVM		15–20 μm	beams	70–100 µm		
HTM		70–130 µm			∼4 kPa (elastic modulus)	
					515 kPa (tensile testing)	
JCT-like	Photolithography (SU-8 scaffolds)	20 µm	7 µm	12 µm		[129]
JCT-like	Electrospinning (PCL scaffolds)	20.3 μm	0.770 µm	5.6 μm^2^	0.95 ± 0.05 MPa (elastic modulus)	[136]
JCT-like	Electrospinning (PCL scaffolds)	30.4 µm	0.600 µm	3.3 µm/ 70.9%	Young’s modulus/tensile modulus 5.15 ± 0.55 MPa/0.17 ± 0.02 MPa	[137]
	Cryoelectrospinning (PCL scaffolds)	76.9 µm	0.430 µm	8.5 µm/ 91.9%	0.79 ± 0.24 MPa/0.03 ± 0.01 MPa	
					Compression modulus/tensile	[138]
JCT-like	MEW	125 µm	10.0 µm	86.8%	11.2 ± 3.3 kPa/13.0 ± 1.7 MPa	
CSM-like	(PCL scaffolds)	299 µm	10.2 µm	84.7%	87.9 ± 75.6 kPa/7.2 ± 2.1 MPa	
UVM-like		140 µm	11.8 µm	91.2%	63.8 ± 79.9 kPa/7.2 ± 2.1 MPa	
Full HTM		506 µm	11.9 µm	84.2%	358 ± 235 kPa/6.9 ± 1.1 MPa	
					Elastic modulus/yield stress	[141]
JCT-like	Electrospinning	20 µm	0.770 µm	5.6 µm^2^	0.94 ± 0.05 MPa/2.84 ± 0.20 MPa	
CSM-like	MEW	610 µm	29.1 µm	0.75 mm	0.18 ± 0.01 MPa/0.39 ± 0.03 MPa	
UVM-like	Combined	260 µm	37.5 µm	0.86 mm	0.14 ± 0.01 MPa/0.24 ± 0.04 MPa	
Full HTM	(PCL scaffolds)	510 µm			0.29 ± 0.03 MPa/0.65 ± 0.22 MPa	
JCT-like	MEW	36 µm	8.0 µm	50 µm	6 MPa Young’s modulus	[142]
CSM-like	(PCL scaffolds)	52 µm	10.2 µm	88 µm	23 MPa Young’s modulus	
UVM-like		76 µm	29.2 µm	130 µm	17 MPa Young’s modulus	
Full HTM		164 µm	8.0–29.2 µm	50.1–131 µm	23 MPa Young’s modulus	

To address this issue, Bikuna-Izagirre et al. combined MEW with electrospinning to recreate the zoning and porosity gradient of the HTM, using electrospun nanofibrous PCL scaffolds to mimic the outmost JCT layer, followed by a MEW-printed middle CSM-like stack and subsequent MEW-printed inner UVM-like stack (Figure 7c) [141]. HTM cells grown on individual or full stacks exhibited high cell viability (80–90%), nuclear alignment, and differential responses to DEX and netarsudil, facilitating the study of how microarchitecture modulates pharmacodynamics [141].

To better mimic the ECM structure of JCT, CSM, and UVM, Gladysz et al. further refined and printed the multilayered PCL scaffolds using MEW, and then cultured HTM cells on these scaffolds, followed by continuous pressure monitoring during perfusion at a single flow rate of 4 µL/min [142]. 3D-printed scaffolds supported HTM growth and exhibited increased outflow resistance due to cell proliferation while proteomic analysis revealed flow-induced changes in protein expression related to protein synthesis and respiration [142]. Lat-B treatment resulted in decreased pressure values and disrupted actin filaments [142]. This study shows the potential for in vitro glaucoma drug testing.

## 4. Scaffold-Free HTM Spheroids and Organoids

3D HTM spheroids without scaffolding offer a self-supporting system, in which cells secrete and remodel their own matrix and preserve cell–cell interactions, avoiding biases from exogenous materials and allowing for a clear study of physiologically relevant cell contractility and ECM remodeling. In general, HTM spheroids have been developed by culturing HTM cells in hanging drop plates (e.g., Perfecta3D^®^) [143,144] or sphere microplates (e.g., Corning^®^ 96-well Black/Clear Round Bottom Ultra-Low Attachment Spheroid Microplate) [145], using cell culture medium supplemented with 0.25% methylcellulose for 6 days. Extensive studies using 3D HTM spheroids, paired with 2D cultures for barrier function characterization, have highlighted the importance of 3D culture systems for modeling DEX or TGFβ2-induced glaucomatous pathology and for screening therapeutics with translational relevance [78,79,83,146,147].

**Figure 8:**
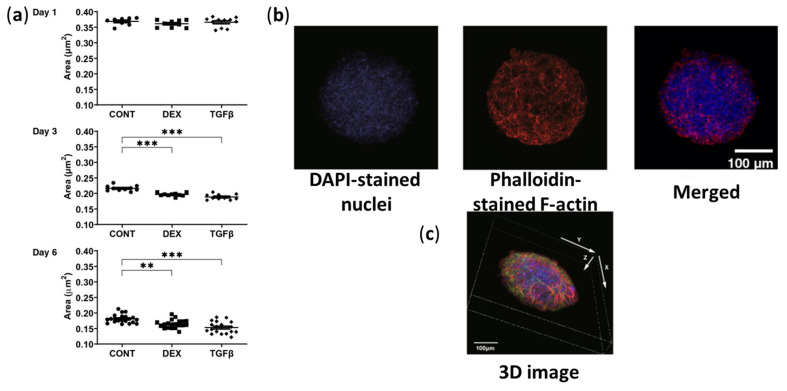
3D HTM spheroids. (**a**) Size analysis of spheroids treated with the vehicle control (CONT), 250 nM DEX, or 5 ng/mL TGFβ2 on days 1, 3, and 6. (**b**,**c**) Representative confocal images of day-6 CONT 3D HTM spheroids at the XY axes plane (**b**) and the 3D XYZ axes volume (**c**). **, *p* < 0.01; ***, *p* < 0.005. Adapted from Watanabe et al., 2021, Sci Rep [144] under the terms of the Creative Commons Attribution (CC BY) 4.0 International License (http://creativecommons.org/licenses/by/4.0/), Copyright © 2021, the authors.

Watanabe et al. demonstrated that culture of ~20,000 HTM cells in hanging drop plates were sufficient to form ~400–500 µm spheroids that responded to 250 nM DEX or 5 ng/mL TGFβ2, developing a clear glaucomatous phenotype [144]. The glaucomatous spheroids demonstrated increased fibronectin, collagen type I (alpha 1 chain) (COL1A1), and α-SMA, reorganization of F-actin into CLANs, nuclear translocation of YAP, a reduction in spheroid size (Figure 8), and an increase in spheroid stiffness [144]. In parallel, paired 2D cultures confirmed DEX/TGFβ2-induced barrier state, showing elevated transepithelial electrical resistance (TEER) and lower permeability after treatment [144]. Ota et al. developed a TGFβ2-induced 3D HTM spheroid model and demonstrated therapeutic effects of ROCK inhibitors in HTM cells by restoring healthy spatial organization and counteracting fibrotic remodeling toward outflow-relevant architecture [143]. Furthermore, the 3D HTM spheroid models were used to test the anti-fibrotic potential of human bone marrow mesenchymal stem cell (MSC)-derived small extracellular vesicles (sEVs) in vitro and in vivo. They demonstrated that culturing and implanting HTM cells with sEVs could significantly mitigate HTM fibrosis by reducing fibronectin and α-SMA expression in 3D HTM spheroids and reducing IOP in mice with chronic ocular hypertension [145].

Overall, HTM spheroids allow the transition to mechanically dependent fibrogenic states to be modeled in a compact and highly reproducible format and are ideal for screening drugs that modulate actin/YAP-TAZ or ECM deposition. Their main limitation is functional―they cannot be perfused nor do they recreate mechanical or porosity gradients comparable to JCT/CSM/UVM. Therefore, they are complementary to, rather than a substitute for, perfusable micropatterned porous scaffolds when the objective is to directly quantify outflow parameters [144]. The scaffold-free spheroid culture system has great potential for HTM organoid generation, particularly when combined with stem cell technologies and adapted for 3D culture assays.

## 5. Clinically Relevant 3D Outflow Pathway Models Using Stem Cell-Derived HTM/HSC Cells

### 5.1. Need for Stem Cell-Derived HTM and HSC Cells

Primary HTM and HSC cells have served as in vitro models for studying aqueous humor outflow and glaucoma pathogenesis. However, primary HTM and HSC cells have limited proliferative capacity and progressively lose their native morphology and gene expression beyond 6–8 passages, developing senescence-associated features. In addition, normal and glaucomatous HTM and HSC cells are derived from different donors, with individual variability that compromises reproducibility, while the scarcity of donor tissues restricts large-scale and longitudinal studies. Stem cell-derived HTM and HSC cells overcome these limitations by providing expandable, genetically stable, and donor-independent cell sources that can be differentiated into defined outflow cell subtypes under controlled conditions. Their uniform genetic background also enables the generation of isogenic normal and glaucomatous models through targeted gene editing, e.g., myocilin (MYOC), cytochrome P450 family 1 subfamily B member 1 (CYP1B1), and latent transforming growth factor beta binding protein 2 (LTBP2).

### 5.2. Role of Stem Cell-Derived HTM and HSC Cells for TM Function Regulation

Histological studies have revealed that both HTM and TM stem cells (TMSCs) exhibit a decline in cell density with age and in glaucomatous eyes, suggesting that the loss or exhaustion of TMSC function contributes to TM degeneration and impaired aqueous outflow [148,149,150]. Conversely, experimental evidence indicates that supplementing or reactivating TMSCs can reverse these pathological changes. TMSCs can home to damaged TM, differentiate into TM-like cells expressing TM cell markers such as Aquaporin 1 (AQP1) and Chitinase-3-like protein 1 (CHI3L1), and significantly improve outflow facility and reduce IOP in both laser-induced and MYOC mutant glaucoma models, with favorable immunomodulatory and safety profiles [151,152,153,154]. Recent in vitro studies have shown that TMSC-derived sEVs can recapitulate many reparative effects of TMSCs via paracrine signaling [155]. These sEVs (30–200 nm), which carry markers such as syntenin, emilin, and neuropilin, were efficiently internalized by TM cells, promoting their proliferation, migration, and resistance to oxidative stress. Similarly, extracellular vesicles derived from immortalized corneal stromal stem cells were shown to counteract DEX-induced HTM dysfunction by significantly reducing Angiopoietin-like 7 (ANGPTL7) expression, a fibrosis-related gene linked to elevated IOP [156]. Mechanistically, stem cell-mediated TM repair involves both direct cellular replacement and indirect paracrine effects through stromal cell-derived factor 1 (SDF1)/C-X-C chemokine receptor type 4 (CXCR4)-driven homing, integrin α5β1-mediated adhesion, anti-inflammatory signaling, ECM remodeling, and stimulation of endogenous TM proliferation [157,158,159].

Induced pluripotent stem cell-derived TM (iPSC-TM) cells recapitulate the morphology, transcriptional signatures, and physiological responsiveness of native TM cells, and have been shown to restore IOP homeostasis and stimulate endogenous TM regeneration, particularly by activating ATP-binding cassette super-family G member 2-positive (ABCG2+) and Nestin+ subpopulations, which serve as biomarkers of TMSCs, and through gap junction-mediated intercellular communication in human anterior segment perfusion and MYOC mutant mouse models [160,161,162,163,164,165]. However, tumorigenicity remains a concern, requiring stringent purification and non-integrating reprogramming methods.

MSCs, including bone marrow-derived MSCs (BM-MSCs) [166,167,168], adipose-derived stem cells (ADSCs) [158], and human cord blood stem cells [169], exert potent paracrine, anti-fibrotic, and immunomodulatory effects that improve TM phenotype and contractility, and can also be induced toward a TM-like lineage responsive to glucocorticoids. In vivo, MSCs or their hypoxia-induced secretome promoted TM regeneration, activated ciliary progenitors, lowered IOP, and protected RGCs by modulating Akt and anti-fibrotic pathways [166,167,168]. Their autologous accessibility and clinical safety make them an attractive option for early-phase clinical translation.

To summarize current HTM differentiation strategies, we compiled representative TMSC-, ADSC-, and iPSC-based protocols, including MYOC and CHI3L1 expression, differentiation efficiency, and functional validation, in Table 2. Among these reported studies, MSC-derived HTM-like cells generally acquired TM functional properties rapidly after induction, whereas iPSC-derived HTM-like cells often required multi-step lineage specification [158,160,161,163,164,170,171,172,173,174]. Both MSC and iPSC can be induced to differentiate into functional TM cells responsive to DEX treatment.

Safety evidence indicates that MSCs have a very low tumorigenic risk across more than 1000 clinical trials, whereas iPSC-based approaches require careful control of genetic stability and residual pluripotency. Anterior chamber delivery allows physiological targeting of TM along aqueous flow, and adjunctive magnetic nanoparticle guidance may enhance localization [175]. Magnetically steered human ADSCs and iPSC-derived HTM cells have demonstrated efficacy in IOP reduction, with human ADSCs showing greater effectiveness by achieving 27% IOP reduction for 9 months after a single dose of only 1500 human ADSCs, leading to enhanced outflow through the conventional pathway and increased TM cellularity [176].

Future directions emphasize Phase I clinical trials prioritizing autologous ADSCs, while exploring iPSC-derived cells and cell-free secretome or exosome therapies developed under strict good manufacturing practice (GMP) manufacturing and release criteria, supported by long-term structural and biomechanical assessment of TM function. The translational potential of stem cell-derived HTM cells highlights the paramount importance of integrating stem cells into 3D HTM models.

### 5.3. Stem Cell-Derived ACOS for Simulated Outflow Facility

Tian et al. developed a stem cell-based, 3D HTM model by seeding iPSC-derived HTM cells on micropatterned porous SU-8 scaffolds, which exhibited similar cell morphology and phenotype to primary HTM cells grown on these scaffolds [131]. In particular, the 3D iPSC-derived HTM model responded to DEX treatment by inducing CLAN formation, increasing expression of myocilin and ECM proteins such as collagen IV and fibronectin, and lowering simulated outflow facility [131]. The establishment of a stem cell-based ACOS opens a new avenue for using iPSC-derived HTM for both normal and genetic mutation-induced, glaucomatous ACOS pairs for understanding outflow physiology and pathophysiology under well-controlled conditions. The ACOS platform can also be populated with other stem cells, e.g., TMSCs, ADSC-derived HTM cells.

**Figure 9:**
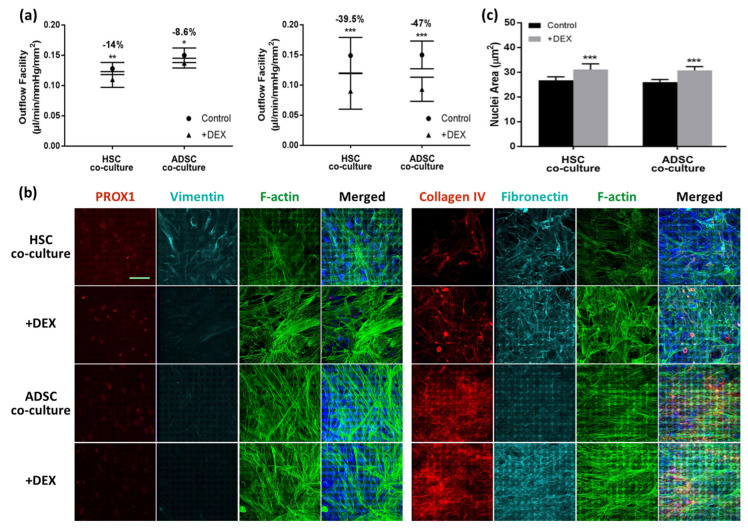
DEX-induced responses to outflow in HTM/HSC and HTM/ADSC-derived HSC-like constructs. (**a**) Simulated outflow facility after 3 and 7 days of treatment with the vehicle control and 100 nM DEX. (**b**) Confocal images of HSC marker and ECM protein expression in the HSC or ADSC-derived HSC-like cell layer of the co-cultured constructs with or without 7-day DEX treatment. Expression of HSC marker PROX1 (**left panel**) and ECM protein of Collagen IV and fibronectin (**right panel**). Constructs were co-stained with vimentin (**left panel**) and F-actin (**right panel**) as well as DAPI to label nuclei. Scale bar = 50 μm. (**c**) Quantification of nuclear size after DEX treatment compared to the control. * *p* < 0.01, ** *p* < 0.001, and *** *p* < 0.0005. Reprinted from Acta Biomater, Vol 105, Tian et al., A bioengineering approach to Schlemm’s canal-like stem cell differentiation for in vitro glaucoma drug screening, 203–213 [132], Copyright (2020), with permission from Acta Materialia Inc., Published by Elsevier Ltd.

With respect to introducing stem cell-derived HSC into an ACOS, Tian et al. first induced ADSCs into HSC-like cells in the presence of vascular endothelial growth factor-C (VEGF-C) and shear stress that is comparable to the level of shear flow in Schlemm’s canal [132]. Then, they co-cultured primary HTM cells and ADSC-differentiated HSC cells in an ACOS, which exhibited a similar simulated outflow facility to a co-cultured primary HTM/HSC ACOS and responded to DEX treatment by increasing ECM protein expression and inducing CLAN formation as well as lowering the simulated outflow facility (Figure 9) [132]. Their work was foundational for developing higher-throughput ACOSs using stem cells to study fundamental outflow physiology and pathophysiology, enable glaucoma drug screening, and support mechanistic studies of outflow regulation.

## 6. HTM Models for Outflow Physiology Studies

### 6.1. Outflow Resistance, Hydraulic Conductivity, and Outflow Facility

Aqueous humor formation follows a circadian rhythm, peaking in the early morning and declining at night, influenced by sympathetic input and circulating catecholamines such as epinephrine [177]. IOP is determined by the dynamic balance between aqueous humor production by the ciliary body (inflow) and the resistance to aqueous humor drainage (outflow). Although theoretically, excessive aqueous humor production could lead to IOP elevation by increasing TM hydraulic load, in practice that is never the case. High pressure glaucoma is characterized by reduced outflow whereas normal tension glaucoma occurs despite outflow remaining within normal limits. Thus, outflow resistance is the primary determinant of IOP, and homeostatic regulation of outflow resistance helps maintain normal IOP; however, increased outflow resistance limits the aqueous humor drainage and underlies the elevated IOP characteristic of glaucoma [177]. Outflow resistance represents the sum of factors that limit the outflow rate, reflecting the extent to which the TM and downstream pathways impede fluid drainage [178].

Outflow facility is the inverse of outflow resistance, which is a functional measure of TM compliance and ease of aqueous humor flows through the TM under a pressure gradient. Low outflow facility corresponds to high outflow resistance and elevated IOP. Outflow facility is defined as a ratio of the outflow rate to the relevant pressure, which is an important indication of outflow resistance in the ocular outflow pathway. The modified Goldmann equation (Equation (1)) describes how IOP is determined by aqueous humor dynamics, and its derived form (Equation (2)) is used to quantify outflow facility, where *C* is outflow facility, *IOP* is the intraocular pressure, *F_in_* is the aqueous production rate, *Fu* is the uveoscleral outflow rate, and *P_v_* is the episcleral venous pressure.

(1) $$IOP=\frac{F_{in}-F_{u}}{C}+P_{v}$$

(2) $$C=\frac{F_{in}-F_{u}}{IOP-P_{v}}$$

In most ex vivo or in vitro perfusion studies of the conventional outflow pathway, there is neither episcleral venous pressure (*P_e_* = 0) nor uveoscleral outflow (*F_u_* = 0). Therefore, the Goldmann equation can be simplified as Equation (3) [34].

(3) $$C=\frac{F_{in}}{IOP}$$

In ex vivo or in vitro perfusion experiments, the perfusion flow rate (*F*, µL/min) is considered equivalent to *F_in_*, the aqueous production rate, and the pressure (*P*, mmHg) in the anterior chamber or perfusion system serves as an analog for IOP, and therefore, we get Equation (4) for calculating outflow facility (*C*, µL/min/mmHg).

(4) $$C=\frac{F}{P}$$

In ex vivo organ culture, outflow facility can be determined using constant flow perfusion [179,180] or 1-level constant pressure perfusion [181], where Equation (4) can be used to calculate outflow facility with the assumption of zero flow at zero pressure.

Additionally, 2-level constant pressure perfusion is commonly used to calculate outflow facility through Equation (5), where *F*_1_ or *F*_2_ represent the flow rate measured at a low perfusion pressure (*P*_1_) or high perfusion pressure (*P*_2_) [182,183]. Tian et al. showed that outflow facility values differed across different perfusion techniques (constant flow perfusion vs. 1-level vs. 2-level constant pressure perfusion) and calculation methods (Equation (4) vs. Equation (5)) [184].

(5) $$C=\frac{F_{2}-F_{1}}{P_{2}-P_{1}}$$

To obtain a more robust and reliable measurement of outflow facility, multi-level constant pressure perfusion can be performed, in which the eye or anterior segment is perfused at multiple precisely controlled pressure levels, and the resulting flow rate at each pressure is measured after the flow is stabilized, fitting a lineal flow rate–pressure relationship [185]. The outflow facility is determined by the slope of the flow rate vs. pressure plot. It is assumed that the outflow facility is pressure-independent, which is usually valid in the physiological pressure range. For example, flow rate vs. pressure plots exhibit linearity up to 25 mmHg for mouse eye, with the linear trend becoming more apparent when the flow rate is measured using more pressure points within a narrow range [186]. To capture pressure-independent outflow facility, a non-linear, power law model is proposed for multi-level constant pressure perfusion of the mouse eye as well [185].

Alternatively, outflow facility can also be measured using multiple flow rate infusions, in which the eye or anterior segment is perfused sequentially at a series of flow ratesand the stabilized pressure at each flow rate is recorded. The outflow facility is calculated as the reciprocal of the slope of the respective pressure vs. flow rate plot [187].

As shown in Table 3, perfusion of ex vivo human anterior segment organ culture offers physiologically relevant systems to study outflow facility and IOP regulation, providing valuable insights into HTM physiology. However, ex vivo organ culture has limitations, such as donor variability, limited tissue viability, and technical challenges in maintaining perfusion stability [34]. ACOSs, in contrast, provide a platform that recapitulates JCT-like behaviors and permits monitoring of both the HTM phenotype and simulated outflow facility, which is determined by measuring pressure during constant flow perfusion at a series of flow rates, with high-throughput and reproducibility [129,130,131,132,133,134].

Outflow facility quantifies how easily aqueous humor exits the eye through the conventional outflow pathway, which is the most physiologically relevant metric of HTM function and a gold-standard functional readout for glaucoma research. It describes how HTM regulates fluid flow under pressure. Simulated outflow facility measured using in vitro models reflects their ability to recapitulate functional performance of HTM as an IOP regulator, pressure-dependent resistance, and dynamic response to stimuli (e.g., pharmacological agents, oxidative stressor, and elevated pressure).

In addition to outflow facility, hydraulic conductivity is used to quantify the permeability of the HTM or HSC cells grown on a filter membrane or insert [188,189,190,191,192,193]. Hydraulic conductivity describes how readily a fluid (e.g., medium, perfusate, and aqueous humor) passes through a porous material (e.g., filter, TM cell monolayer grown on the filter, and TM tissue) under pressure. Unlike outflow facility, hydraulic conductivity reflects only the intrinsic permeability of the HTM and is independent of the overall outflow pathway. For HTM or HSC cells grown on a filter membrane, hydraulic conductivity (*L_p_*, μL/min/mmHg/cm^2^) can be calculated by Equation (6), where *F* (μL/min) is the perfusion flow rate, *P* (mmHg) refers to the pressure in the perfusion system, and *A* (cm^2^) represents the filter surface area.

(6) $$L_{p}=\frac{F}{P\times A}$$

### 6.2. Bioengineered ACOS for Simulating Outflow Facility Responses to IOP-Modulating Agents

**Figure 10:**
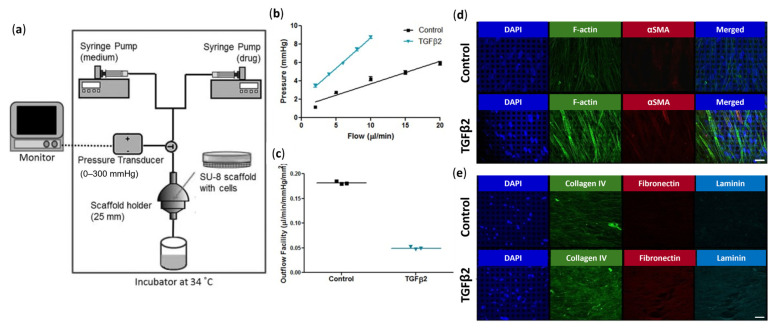
The ACOS made of primary HTM cells on micropatterned porous SU-8 scaffolds can be used to test the effect of the IOP-modulating agent, such as TGFβ2 on outflow function. (**a**) Schematic perfusion setup using ACOS perfused with the vehicle control or 2.5 ng/mL TGFβ2 for 9 days. (**b**) The graph of pressure vs. flow rate that allows to calculate the simulated outflow facility. The ACOS was perfused in an apical-to-basal direction with perfusate at various rates for 6 h per flow rate to ensure it reached a steady pressure, and the pressure was continuously monitored and recorded. (**c**) Calculated simulated outflow facility of the ACOS treated with the vehicle control (square) or TGFβ2 (triangle), demonstrating increases in outflow resistance in 3D HTM cultures after a 9-day treatment with TGFβ2. (**d**,**e**) Confocal images of the HTM cell-scaffold constructs demonstrated enhanced cyto-skeletal F-actin rearrangement and αSMA expression (**d**), as well as induced expression of ECM proteins, including collagen IV (green), fibronectin (red), and laminin (cyan) (**e**). Scale bar = 30 μm. (**a**) Reproduced with permission from Torrejon et al., 2013, Biotechnol. Bioeng. [129], Copyright © 2013 Wiley Periodicals, Inc. (**b**–**d**) Reproduced from Torrejon et al., 2016, Sci Rep [134], under a Creative Commons Attribution (CC BY) 4.0 International License (http://creativecommons.org/licenses/by/4.0/), Copyright © 2016, the authors.

Torrejon et al. integrated the SU-8 scaffold-based 3D HTM model into a perfusion platform, developing an ACOS that permits monitoring of simulated outflow facility [129]. The confluent HTM cell–scaffold construct was securely placed in the perfusion chamber and perfused with the basal media, vehicle control, or media with IOP-modulating agents in an apical-to-basal direction at a series of flow rates (2, 4, 6, 8, and 16 μL/min [131,132], or 2, 10, 20, and 40 μL/min [129,133,134]), for 6 h per flow rate to ensure each flow reaches steady state. Pressure was continuously monitored and recorded using pressure transducers (Figure 10a) [129]. After perfusion, the graph of pressure vs. flow rate was plotted and its slope was determined by linear regression (Figure 10b). The outflow facility (µL/min/mmHg) of the ACOS was calculated as the inverse of the slope of the pressure vs. flow rate plot and normalized to the perfusion surface area (µL/min/mmHg/mm^2^) (Figure 10c) [134]. 

The HTM-only ACOS provided resistance to flow in the same range as JCT in vivo [129]. Furthermore, the HTM-based ACOS exhibited the expected response to steroid treatments (e.g., PA, DEX), showing increased outflow resistance and reduced outflow facility, validating the utility of the ACOS for evaluating pharmacologic agents [131,133]. Treatment of the ACOS with IOP-elevating agents, such as TGFβ2, decreased outflow facility, induced F-actin stress fiber rearrangement, and enhanced ECM protein expression (Figure 10d) [134]. In contrast, treatment of the ACOS with an IOP-lowering agent, such as Rho-associated protein kinase (ROCK) inhibitor Y27632, increased outflow facility and reversed the TGFβ2-induced effects, returning outflow facility to the near-control level during co-treatment [134]. These results confirmed the utility of the ACOS for evaluating pharmacologic agents and elucidating their mechanisms of IOP modulation.

Tian et al. further demonstrated the establishment of a 3D HTM/HSC ACOS, using primary HTM co-cultured with primary or stem cell-derived HSC cells on SU-8 scaffolds, for monitoring simulated outflow facility by treatment with DEX, which decreased outflow facility [132]. These findings validate the ACOS for testing IOP-modulating agents and demonstrate the feasibility of generating steroid- or TGFβ2-induced 3D glaucomatous models for concurrently assessing altered glaucomatous cell phenotype, abnormal ECM accumulation, and reduced simulated outflow facility.

The significance of these clinically relevant, normal and glaucomatous, 3D HTM or 3D HTM/HSC systems has been further confirmed by industrial collaborations that utilize the ACOS in vitro to measure simulated outflow facility (Table 4) as an essential complementary approach to animal studies of potential outflow regulators (e.g., ROCK inhibitor―Y27632 [134], transient receptor potential vanilloid 4 (TRPV4) antagonist―HC-067047 [195,196], adenosine A1 receptor agonist―trabodenoson [197], nitric oxide donor―NCX 667 [198], nitric oxide-donating bimatoprost―NCX 470 [199], and ANGPTL7 and its blocking antibody [200]), in which in vitro ACOS results were consistent with in vivo animal studies.

### 6.3. Limitations of PCL Scaffold-Based HTM Models for Perfusion Studies

Microfabricated, well-defined, porous PCL scaffolds show great potential to recapitulate JCT, CTM, and UVM architecture, which support HTM cell growth, phenotype, and disease modeling. However, current PCL-based HTM models have not demonstrated outflow facility assessment of the cell–scaffold construct although outflow facility of the acellular PCL scaffold was measured under constant flow perfusion at a series of flow rates (10, 20, 40, 80, and 160 µL/min) [136]. The perfusion flow rate reached as high as 160 µL/min, suggesting these PCL scaffolds might be too porous to generate measurable changes in pressure at a low flow rate. These high flow rates are neither physiologically relevant nor practical for perfusing the cell–scaffold constructs. Therefore, drug responses to DEX and netarsudil were assessed by perfusing the HTM cell–scaffold construct at a single flow rate (20 μL/min), and only the transmembrane pressure was reported for groups with or without drug treatments [136]. While MEW-printed PCL scaffolds could recapitulate all three HTM layers (JCT, CSM, and UVM) and permit perfusion at a physiologically relevant flow rate (4 µL/min), this 3D HTM model was limited to measuring pressure changes over time at a single flow rate and faced challenges in maintaining stable flow due to clogging and air-bubble formation during longer-term culture [142].

If a 3D HTM model could measure transmembrane pressure over time only at a single flow rate, or it could not maintain stable flow to reach steady-state pressure at each flow rate, it would be unable to calculate outflow facility (Table 3). Continued refinement and optimization of the scaffold architecture may improve flow stability and allow pressure measurements across multiple flow rates near the physiologically relevant range, enabling calculation of outflow facility, which will facilitate assessing pressure-dependent resistance, fully characterizing drug response, distinguishing structure vs. functional changes, or modeling dynamic HTM physiology.

### 6.4. Traditional HTM Cell Monolayer Culture on Filter Membranes for Perfusion Studies to Measure Hydraulic Conductivity

Prior to the development of 3D HTM models, Perkins et al. laid foundational work by using commercially available filter membranes or inserts for culturing HTM cells and for measuring hydraulic conductivity in a perfusion setup [188]. Filter-based HTM cell cultures were perfused under constant pressure [188,189,190] or at a constant flow rate [193]. As shown in Table S2, HTM or HSC cells grown on filters (e.g., Millicell filters, Snapwell inserts) were used to study the effect of laser irradiation on hydraulic conductivity [189] and the effects of pharmacological agents on the hydraulic conductivity, including cell-permeable mycotoxin—cytochalasin B [188], β-adrenergic agonist—isoproterenol [191], adrenergic agonist—epinephrine [190], calcium-chelating agent—Na_2_EDTA [192], and IOP-elevating steroid—DEX [190,193], as well as effects of hydrostatic pressure gradients and pharmacological agents on permeability [192]. For example, perfusion studies of HTM cells grown on 0.4 µm pore size filter inserts demonstrated that hydraulic conductivity of a monolayer of HTM cells can be enhanced through the application of non-destructive, low fluence diode laser irradiation [189].

In a typical constant pressure perfusion setup, fluid flows from an elevated medium reservoir through a flowmeter that measures the flow rate, then through an in-line pressure transducer or sensor that monitors pressure, and finally into the perfusion chamber that hosts the HTM or HSC cell monolayer grown on a filter, with effluent collected in a container. Alternatively, perfusion can be conducted at a constant flow rate (i.e., 20 μL/min) using a syringe pump while pressure is measured with a pressure sensor [193]. In both configurations, hydraulic conductivity can be calculated using Equation (6).

The filter-based HTM culture system provides a useful in vitro perfusion platform to study the regulation of aqueous outflow. However, there are some limitations. First, these filters are track-etched membranes and possess irregular pore structures, which may limit their ability to support in vivo-like HTM cell growth [129]. Second, some monolayers cultured on filters for three weeks at confluence still lacked mature intercellular junctions, as evidenced by low TEER, which cannot withstand control chamber exchanges and 5 mmHg pressure gradients in the perfusion experiment [192]. Third, although monolayers cultured on filters for more than five weeks exhibited higher TEER, they consistently detached from filters during control chamber exchanges [192], limiting their ability for long-term cell culture and perfusion experiments. Fourth, although it provides a basic functional readout of HTM permeability and allows for comparison across experimental conditions, it only mimics a “snapshot” of outflow function.

Measuring hydraulic conductivity assesses the intrinsic permeability of HTM constructs, whereas simulated outflow facility evaluates their functional ability to regulate pressure-dependent aqueous humor drainage. Together, they provide complementary structural and functional validation of 3D HTM models.

### 6.5. 3D HTM/HSC Cultures in Microfluidic Devices for Assessment of Outflow Rate

**Figure 11:**
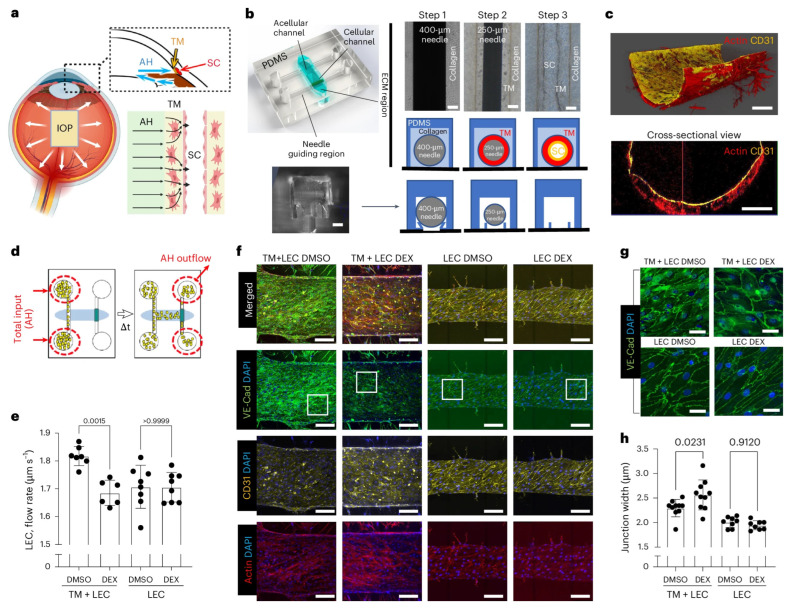
The multicellular human ocular fluid outflow on-chip recapitulates steroid-induced glaucoma in vitro. (**a**) Schematics of IOP regulation by aqueous humor (AH) outflow through the TM and SC. White arrows indicate direction of pressure and black arrows indicate direction of AH flow. (**b**) Schematics of the microfluidic device and steps for generating the TM–SC double-layered channel structure. (**c**) Immunostaining of HTM cells and LECs in the device (red, phalloidin-stained F-actin; yellow, CD31). (**d**) Schematics to determine the average outflow rate. (**e**) Average flow rate after treatment with 1 μM DEX for 7 days in TM + LEC co-culture or LEC monoculture in the ocular fluid outflow on-chip. (**f**) VE-cadherin/CD31/actin staining before and after DEX treatment (1 μM) in the TM + LEC co-culture or the LEC monoculture. The white box outlines the region corresponding to zoomed images of VE-cadherin shown in (**g**). (**h**) LEC junction widths before and after DEX treatment. Scale bars: 50 μm (**b**,**c**), 100 μm (**f**), and 25 μm (**g**). Reproduced from Lu et al., 2025, Nat. Cardiovasc. Res. [194], under a Creative Commons Attribution–NonCommercial–NoDerivatives (CC-BY-NC-ND) 4.0 International License (http://creativecommons.org/licenses/by-nc-nd/4.0/), Copyright © 2025, the authors.

Lu et al. developed a biomimetic microphysiological system―human ocular fluid outflow on-chip―using a polydimethylsiloxane (PDMS) microfluidic device with two parallel channels (one acellular and one cellular), embedded within a 3D collagen/ECM region (Figure 11) [194]. This seminal work introduced the first human outflow on-chip system capable of revealing TM-mediated SC dysfunction in steroid-induced glaucoma, fundamentally advancing both mechanistic understanding and bioengineered disease modeling.

In this study, an M-shaped needle guide enabled layering of HTM cells and lymphatic endothelial cells (LECs, mimicking HSCs) by sequentially inserting needles of different diameters to mimic the aqueous humor drainage pathway. Although it was not used to measure the simulated outflow facility, the outflow function was evaluated by measuring the outflow rate (i.e., average flow velocity) under an elevated hydraulic pressure. Upon treatment with DEX, the model exhibited decreased fluid outflow and tightened endothelial junctions, validating its usefulness to mimic steroid-induced glaucoma. Mechanistically, the outflow dysfunction was linked to ALK5/VEGF-C signaling, highlighting HTM-mediated regulation of SC endothelial behavior [194]. This platform provides an alternative model system to bridge in vitro and in vivo models for studying outflow pathophysiology and screening targeted therapeutics for glaucoma.

The organ-on-a-chip platform provides a promising alternative to study aqueous humor hydrodynamics [201]. The current study measures the outflow rate at a single pressure [194], providing a simple, useful measure of HTM permeability. However, it has not captured pressure-dependent behavior or outflow facility yet. Further development of the fluid outflow on-chip platform that allows for measuring the outflow rate at a series of elevated hydraulic pressures will enable calculation of the simulated outflow facility, reflecting dynamic HTM physiology.

## 7. Summary and Future Outlook

In vitro tissue models are useful for preclinical drug testing to improve the clinical translation rate of therapeutic candidates. While 2D models offer the advantage of being a simplistic platform to assess drug/therapeutic toxicity through high-throughput studies, and animal models offer the advantage of evaluating whole organism drug interactions and immune reactions, their physiological and species relevance limits their reliability for drug efficacy and mechanism of action studies. 3D HTM models that enable high-throughput, outflow-functional testing of therapeutic candidates, provide disease-relevant endpoint assays for evaluating therapeutic efficacy, and accurately recapitulate HTM physiology, including tissue architecture, cellular phenotype and function, biomechanical and hydrodynamic characteristics, and drug responsiveness of HTM tissue, are essential for identifying therapeutic candidates with strong translational potential. Existing 3D HTM models have been able to recapitulate certain aspects of HTM physiology (Table 5).

These 3D HTM models, however, have some limitations, such as donor variability, long-term stability, reproducibility, and scalability (Table 5). While donor–donor variability is an important feature that reflects differences in therapeutic responses observed in the human population, such variability can obscure therapeutic potential in in vitro studies due to inconclusive results across donor tissues. To address this challenge, power analysis may be employed to determine appropriate sample sizes, along with rigorous experimental design and statistical approaches. Alternatively, the use of stem cell-derived HTM and HSC cells offers well-defined, condition-controlled model systems that further reduce variability.

As far as long-term stability is concerned, scaffold-free spheroids or organoids tend to increase in size over time, leading to necrotic core formation or fusion of organoids. These issues can be mitigated by dynamic cultures that enhance oxygen and nutrient transport while avoiding fusion. Hydrogel-based HTM cultures may experience hydrogel degradation and diffusion limits over time; stimulating HTM cells to deposit their own ECM, combined with dynamic cultures, can improve their stability. In contrast, synthetic scaffolds (e.g., SU-8, PCL) and microfluidic chips inherently provide long-term structural stability. Therefore, the combination of organoids and hydrogel-based HTM cultures with synthetic scaffolds or microfluidic devices may offer a robust, stable, and perfusable platform for studying TM biology and physiology.

Similarly, 3D-printed scaffolds, microfabricated scaffolds, and microfluidic devices exhibited high reproducibility due to robust and standardized microfabrication protocols. In contrast, scaffold-free organoid and hydrogel-based HTM cultures show lower reproducibility due to batch-to-batch variation; however, using multi-well culture platforms can help mitigate the issue. Overall, further standardization across these HTM culture systems will enhance reproducibility.

Regarding scalability, scaffold-free organoids and hydrogel-based HTM cultures are relatively easy to scale up due to their accessibility and compatibility with high-throughput formats. In contrast, the need for 3D printers, electrospinning equipment, or cleanroom and microfabrication facilities limits the scalability of microfluidic devices and scaffold-based HTM cultures. Developing cleanroom-independent microfabrication approaches and simplifying microfluidic fabrication protocols could substantially improve their accessibility and scalability.

Furthermore, developing a standardized validation framework will increase the fidelity and translational value of bioengineered HTM models. Such a framework should include 3D morphological characterization, assessment of characteristic marker expression and ECM deposition, evaluation of cell contractility and mechanical properties, measurement of simulated outflow facility, and analysis of steroid-induced drug responses.

The integration or combination of these 3D HTM models, for example, using hydrogels to recapitulate biomechanics, microfluidics to simulate hydrodynamics, and micropatterned porous SU-8 or a 3D-printed PCL scaffold-based system to enable outflow facility measurement, would generate a more powerful in vitro HTM outflow platform for physiology and pathophysiology studies in glaucoma. Future advances in photolithography, soft lithography, and 3D printing will make it possible to produce perfusable and mechanically stable, ECM-mimicking scaffolds that support HTM and HSC cell growth and allow us to study outflow facility and IOP regulation under physiologically relevant outflow parameters.

As recommended by Johnson et al., physiologically relevant parameters (Table 6) should be considered in experimental and mathematical modeling of the trabecular outflow pathway [202]. Furthermore, integrating stem cells with 3D HTM models provides a promising avenue for establishing well-controlled, clinically relevant 3D outflow pathway systems. As outlined by Dautriche et al., bioengineered 3D HTM outflow models should be evaluated in terms of HTM phenotype (e.g., characteristic, confluent, and permeable TM complex), physiological function (e.g., phagocytic activity, constitutive chemokine secretion, and in vivo-like outflow facility), and cellular responses to established IOP-elevating agents (e.g., DEX-induced increase in myocilin expression and ECM accumulation accompanied by reduced outflow facility) and potential IOP-lowering agents (e.g., increase in outflow facility) [203].

In addition to facilitating discovery of new IOP-drug targets and high-throughput drug screening and therapeutic testing, existing, integrated, or future 3D HTM models should be built on adaptable 3D culture systems that permit the testing of emerging cell and gene therapy modalities to advance personalized treatment options. Furthermore, these models need to support the development of 3D culture-compatible high-throughput assays. In addition to traditional gene, protein, or secretome biomarker evaluation, these 3D HTM models need to permit outflow facility measurement and IOP-modulation assessment to improve the predictive validity of TM-targeted therapies.

We envision several future research directions in bioengineering HTM for outflow physiology and glaucoma research, including but not limited to:

- Development of advanced multicellular co-culture systems that more accurately recapitulate the anatomy of the conventional outflow pathway.
- Use of patient-specific, stem cell-derived HTM and HSC models.
- Advancement of HTM models that replicate chronic ECM remodeling and aging.
- Utilization of dynamic, pressure-controlled or flow-controlled hybrid scaffold-based or microfluidics-based platforms for evaluation of the HTM phenotype and simulated outflow facility.
- Integration of omics with outflow physiology to elucidate mechanisms underlying HTM function.
- Implementation of high-throughput drug screening with respect to changes in TM biomechanics and simulated outflow facility to reveal mechanisms of IOP-modulation and identify therapeutic targets.
- Development of biomimetic, implantable, and regenerative scaffolds.
- Standardization, benchmarking, and validation against ex vivo, preclinical, and clinical data.
- Data integration and AI-enabled functional modeling.

Overall, these models should provide a human-relevant rigorous and high-throughput drug testing platform for preclinical research before the optimization of formulations for administration in vivo in animal studies. Such 3D HTM models would not only improve the predictive efficacy and reliability of preclinical research studies, but also reduce the burden on animal testing, in alignment with the latest FDA Modernization Act 2.0.

## Figures and Tables

**Table 2:** Differentiation paradigms for generating HTM-like cells from MSCs and iPSCs and their functional characterization.

Cell Source	Strategy	Duration	MYOC	CHI3L1	Key TM Markers Reported	Differentiation Efficiency	Functional Validation	Refs.
Human TMSC	SP/clone isolation → AH or FBS	≤10 d	DEX-inducible	Inducible	AQP1, MGP, TIMP3	NQ	Phagocytosis; TM marker induction	[170]
Human ADSC	TM-ECM + TM-CM or co-culture TM induction	~10 d	DEX-inducible	Positive	AQP1, CHI3L1	Phagocytosis-positive cells ~65–86%	Phagocytosis; DEX response (MYOC, CLAN formation)	[158]
Mouse iPSC	Co-culture with human TM cells (HTM-5; Transwell inserts) → TM-like induction	7–21 d	DEX-inducible	NR	CAV1, COL4A5, MGP, TIMP3, VCAM1, MYOC	NQ	Phagocytosis; MMP3 secretion; DEX-induced MYOC secretion	[171]
Mouse iPSC	TM-CM media–induced TM-like differentiation	~7–14 d	DEX-inducible	NR	LAMA4, TIMP3;	NQ	CLAN formation; TM-like phenotype; TM proliferation support (Cx43-dependent)	[161,164]
Human iPSC	Co-culture with primary TM cells	~60 d	Positive	Positive	Vimentin, AQP1, MGP, COL I/IV, TIMP3	NQ	Phagocytosis; transcriptomic similarity to TM	[163]
Human iPSC	Two-step: iPSC → NCCs (NGFR/HNK1+) → TM induction on TM-ECM + TM-CM	NCCs ~10 d; TM ~10–14 d	DEX-inducible	Positive (DEX-responsive)	CHI3L1; DEX ↑MYOC, ↑ANGPTL7	NCC induction efficiency ~85% (NGFR^+^)	DEX response (MYOC, ANGPTL7); CLAN formation	[172]
Human iPSC	EB differentiation on TM-ECM + TM-CM	~30 d	NR	Positive	CHI3L1, WNT1, α3-integrin, AQP1	NR	Phagocytosis	[160]
Human iPSC	Two-stage cytokine-driven TM-like differentiation (xeno-free)	Stage1: 7 d; Stage2: 14 d	DEX-inducible	NR	LAMA4, TIMP3, AQP1, MYOC, COLIV	NQ	CLAN formation; TM-like morphology; transcriptomic similarity to TM	[173,174]

Note: AH, aqueous humor; CM, conditioned medium; EB, embryoid body; ECM, extracellular matrix; NCCs, neural crest cells; NR, not reported; NQ, not quantified; SP, side population; →, directed differentiation step; and ↑, increase.

**Table 3:** Overview of HTM models for perfusion studies.

Model	Cells/Components	Matrix/ Material	Flow and Mechanical Control	Primary Readouts	Advantages	Limitations	Refs.
**Measurement of outflow facility**							
Ex vivo anterior segment perfusion	Whole anterior segment (donor)	Native corneal/limbal tissue ECM	Constant flow perfusion or constant pressure perfusion	Whole-tissue outflow facility, IOP response, histology	Closest to the in vivo physiology	Low throughput; donor variability; tissue availability	[34]
ACOS	Primary and stem cell-derived HTM and HSC cells	Micropatterned porous SU-8 scaffolds in perfusion chamber	Constant flow perfusion at 2–16 or 2–40 µL/min while monitoring pressure	Simulated outflow facility, cell phenotype, drug response	Reflecting pressure-dependent outflow resistance and dynamic HTM physiology; high throughput; high producibility; consistent; perfusion ready	Cleanroom needed for fabrication; non-physiologically relevant elastic modulus of SU-8 scaffolds (much higher than HTM ECM)	[129,131,132,133,134]
Nanofibrous scaffold-based JCT model	Acellular scaffold	Electrospun nanofibrous PCL scaffolds in perfusion chamber	Constant flow perfusion at 10, 20, 40, 80, and 160 μL/min while measuring pressure	Simulated outflow facility of the scaffold alone	Design flexibility; perfusion ready	Elastic modulus of PCL scaffolds higher than HTM ECM; high flow rates not suitable for cell perfusion	[136]
**Measurement of hydraulic conductivity**							
Filter membrane-based perfusion	HTM cells HSC cells HSC cells Fetal HTM Adult HTM	0.45 µm Millicell filters 0.45 µm Millicell filters 0.4 µm Snapwell filter 0.45 µm Millicell filters	Constant pressure perfusion at 5 mm Hg; under an elevated pressure with 0.5 mmHg/µL/min resistor; at 6 mmHg with gradual decrease; constant flow perfusion at 20 µL/min	Hydraulic conductivity, drug response	Easy access; perfusion ready	Limiting to a basic permeability readout rather than a full functional assessment of HTM outflow behavior; lack of 3D ECM context; mismatched stiffness and pore size with HTM	[188,189,190] [191] [192] [193]
**Measurement of outflow rate**							
Ocular fluid outflow on-chip	HTM cells	Microfluidic PDMS chip with 2-parallel cylindrical microchannels in collagen hydrogel	Constant pressure perfusion under elevated hydraulic pressure	Average flow velocity (i.e., dividing total outflow volume by duration of time)	Physiologically relevant ECM mimics and flow velocity	Complicated fabrication protocol; lack of outflow facility measurement	[194]
**Demonstration of pressure-dependent outflow resistance (pressure vs. time graph) at only one single flow rate**							
Hydrogel-based HTM model	HTM cells	Lyophilized collagen–GAG scaffolds	Constant flow perfusion at 61.09 µL/min	Pressure vs. time, cell phenotype, drug response	ECM mimics biochemically and biomechanically	Flow instability; inability to calculate outflow facility	[109]
	Primary HTM cells	MAX8B peptide hydrogels	Constant flow perfusion at 3 µL/min				[119]
Multilayer HTM model	Primary HTM cells	MEW-printed PCL stacks	Constant flow perfusion at 4 μL/min		Mimicry of JCT, CSM, and UVM		[142]

**Table 4:** Representative ACOS perfusion studies using SU-8 scaffolds.

3D Model	Treatment	Duration	Outflow Facility (μL/min/mmHg/mm^2^)	Reference
3D HTM cell–scaffold constructs				
Primary HTM cells grown on SU-8 scaffolds for 14 days	Vehicle control 300 nM PA	>7 days	0.131 ± 0.003 0.093 ± 0.004	[133]
	Vehicle control 2.5 ng/mL TGFβ2 10 µM Y27632 TGFβ2 + Y27632	9 days	~0.17 ~0.02 ~0.24 ~0.16	[134]
	Vehicle control 25 nM GSK101 1 μM HC-067047	24 h	~0.25 ~0.18 ~0.4	[195]
Primary HTM cells grown on SU-8 scaffolds for 12–14 days, treated with 500 nM DEX for 3 days (3D glaucomatous model)	Vehicle control 1% PA 1% PA + 1 µm HC-067047 (TRPV4 antagonist)	6 days	0.32–0.37 0.08–0.12 0.45–0.66	[196]
3D HTM/HSC cell–scaffold constructs				
Primary HTM cells grown on SU-8 until confluency and co-cultured with HSC cells for 7–10 days	Vehicle control 500 nM DEX 0.56 nM ANGPTL7 1.1 nM ANGPTL7 3.3. nM ANGPTL7	9 days	0.35–0.90 0.075–0.18 0.51–1.0 0.26–0.74 0.16–0.63	[200]
Primary HTM cells grown on SU-8 until confluency and co-cultured with HSC cells for 10 days, and then treated with TGFβ2 (2.5 ng/mL) for 6 days (3D glaucomatous model)	Vehicle control 100 µM NCX 667	Perfused for 20 h (5 h at each of 4 flow rates)	0.08 ± 0.04 0.31 ± 0.10	[198]
Primary HTM cells grown on SU-8 until confluency and co-cultured with HSC cells for 10 days, and then treated with TGFβ2 (5 ng/mL) for 6 days (3D glaucomatous model)	Naive (no TGFβ2 pretreatment) Vehicle control 10 µM NCX 470 10 µM bimatoprost	Perfused for 20 h (5 h at each of 4 flow rates)	0.73 ± 0.04 0.47 ± 0.02 0.76 ± 0.03 0.67 ± 0.04	[199]

**Table 5:** Summary of existing 3D HTM models to recapitulate HTM physiology.

Key Features	Scaffold-Free Organoid	Hydrogel-Based	SU-8 Scaffold-Based	PCL Scaffold-Based	Outflow On-Chip
Tissue Architecture	JCT-like	JCT	JCT	JCT/CSM/UVM	JCT
Cellular Phenotype and Function					
- Marker expression	Yes	Yes	Yes	Yes	Yes
- ECM deposition	Yes	Yes	Yes	Yes	
- Cytoskeletal organization		Yes	Yes	Yes	Yes
- Phagocytic activity		Yes	Yes		
- Steroid responsiveness		Yes	Yes	Yes	Yes
- Regulation of ECM turnover	Yes		Yes		
Biomechanics					
- ECM composition		Yes	Gelatin-coated	Gelatin-coated	collagen
- Stiffness	Yes	Yes			
- Mechanosensitivity	Yes	Yes			
- Contractility	Yes	Yes			
Hydrodynamics					
- Outflow facility			Yes [131,132,133,134]	Scaffold only [136]	
- Pressure-dependent outflow resistance			Yes		
- Hydraulic conductivity			Yes [130]		
- Outflow rate					Yes [194]
HTM-HSC interaction			Yes [132]		Yes [194]
Limitations	Scaffold-free organoid	Hydrogel-based	SU-8 Scaffold-based	PCL Scaffold-based	Outflow on-chip
- Long-term stability	+	++ to +++	++++	++++	++++
- Reproducibility	+	++	+++++	++++	++++
- Scalability	+++++	++++	+++	+++	++

Note: Limitations are rated on a scale from + to +++++, where +++++ represents the highest degree of the feature and + the lowest (i.e., the most limitation).

**Table 6:** Parameters defining outflow through the trabecular meshwork and inner wall of Schlemm’s canal.

Parameters	Estimated Values	
Outflow rate (*Q*)	2.5 µL/min [204]	
Flow velocity through TM (*v*)	10 µm/s [202]	*v* = *Q*/(π *d* *h* ε)
HTM dimensions		
Average height in the anterior–posterior direction (*h*) Internal limbal diameter (*d*)	300 µm [205] 11.6 mm [205]	
Circumference (π *d*)	36.4 mm [205]	
Porosity (ε)	37.4 ± 5.9% [206]	
Flow velocity through the inner wall of SC (*v*)	~4000–5000 µm/s [202]	*v =* 4 *Q*/(*n* *A_iw_* π *D*^2^)
Inner wall pore density (*n*)	835 pores/mm^2^ [207]	
Inner wall area (*A_iw_*)	11 mm^2^ [208]	
Pore diameter (*D*)	1.2 µm [202] (0.5–1.5 µm) [209]	
Hydrodynamic shear stress (*τ*)	0.01 Pa [202]	*τ = µv/D*
Viscosity of aqueous humor (*µ*) Characteristic length scale of the flow (*D*)	7 × 10^−4^ Pa s [202,210] 0.5 µm [202]	

## Data Availability

This study did not generate any new data. All data supporting this review are available in the cited literature.

## Supplementary Materials

The following supporting information can be downloaded at: https://www.mdpi.com/article/10.3390/bioengineering13030291/s1, Table S1: List of pharmacological agents applied in 3D HTM model validation and testing. Table S2: Representative perfusion studies using HTM or HSC cells grown on filters.

## Abbreviations

The following abbreviations are used in this manuscript:

2D	Two-dimensional
3D	Three-dimensional
αSMA	α-smooth muscle actin
ABCG2+	ATP-binding cassette super-family G member 2-positive
ACOS	Artificial conventional outflow system
ADSC	Adipose-derived mesenchymal stem cell
AH	Aqueous humor
AM	Acrylamide
ANGPTL7	Angiopoietin-like 7
AQP	Aquaporin
AV	Aqueous vein
BM-MSC	Bone marrow-derived mesenchymal stem cell
CC	Collector channels
CCEs	Collector channel entrances
CCL	Chemokine
CHI3L1	Chitinase-3-like protein 1
CLAN	Crosslinked actin network
COL1A1	Collagen type I alpha 1 chain
COL4A5	Collagen type IV alpha 5 chain
COLI/IV	Collagen type I and collagen type IV
COLIV	Collagen type IV
CS	Chondroitin sulfate
CSM	Corneoscleral meshwork
CTGF	Connective tissue growth factor
CXCR4	C-X-C chemokine receptor type 4
CYP1B1	Cytochrome P450 family 1 subfamily B member 1
DEX	Dexamethasone
ECM	Extracellular matrix
EDTA	Ethylenediaminetetraacetic acid
EP2	Prostaglandin E2 receptor 2
ER	Endoplasmic reticulum
ERK	Extracellular signal regulated kinase
EV	Episcleral vein
FBS	Fetal bovine serum
FN	Fibronectin
FP	Prostaglandin F prostanoid receptor
GAG	Glycosaminoglycan
GelMA	Gelatin methacryloyl
GMPs	Good manufacturing practices
GTM	HTM cells derived from donors with glaucoma
HA	Hyaluronic acid
HNK1	Human natural killer-1 antigen
HSC	Human Schlemm’s canal
HTM	Human trabecular meshwork
IL	Interleukin
IOP	Intraocular pressure
iPSC	Induced pluripotent stem cell
iPSC-TM	Induced pluripotent stem cell-derived trabecular meshwork
JCT	Juxtacanalicular connective tissue
Lat-B	Latrunculin B
LEC	Lymphatic endothelial cell
LTBP2	Latent transforming growth factor beta binding protein 2
MCP	Monocyte chemoattractant protein
MEW	Melt electrowriting
MGP	Matrix Gla protein
MSC	Mesenchymal stem cell
MYOC	Myocilin
NCCs	Neural crest cells
NGFR	Nerve growth factor receptor
PA	Prednisolone acetate
PCL	Poly-ε-caprolactone/polycaprolactone
PDMS	Polydimethylsiloxane
p-MLC	Phosphorylated myosin light chain
POAG	Primary open-angle glaucoma
RGCs	Retinal ganglion cells
ROCK	Rho-associated protein kinase
ROS	Reactive oxygen species
SC	Schlemm’s canal
SDF1	Stromal cell-derived factor 1
SEM	Scanning electron microscopy
sEVs	Small extracellular vehicles
siC	siRNA control
siRNA	Small interfering RNA
SPARC	Secreted protein acidic and rich in cysteine
STL	Stereolithography
TAZ	Transcriptional co-activator with PDZ-binding motif
TEER	Transepithelial electrical resistance
TGFβ	Transforming growth factor beta
TGM	Transglutaminase
TIMP3	Tissue inhibitor of metalloproteinases-3
TM	Trabecular meshwork
TMSCs	Trabecular meshwork stem cells
TRPV4	Transient receptor potential vanilloid 4
UVM	Uveal meshwork
VCAM1	Vascular cell adhesion molecule 1
VEGF-C	Vascular endothelial growth factor-C
YAP	Yes-associated protein
