# Supplementary material for: Bioengineered 3D Human Trabecular Meshwork Models for Outflow Physiology and Glaucoma Research
Source: Bioengineering (Basel). 2026 Feb 28;13(3):291. doi: 10.3390/bioengineering13030291 (PMC13024433; doi:10.3390/bioengineering13030291)
Supplement: Supplementary file 1 [file bioengineering-13-00291-s001.zip › bioengineering-4134594-supplementary.pdf]

bioengineering

MDPI

Supplementary Materials

Bioengineered 3D Human Trabecular Meshwork Models for Outflow Physiology and Glaucoma Research

Table S1

Table S2

https://doi.org/10.3390/bioengineering13030291

Table S1. Treatments used in 3D HTM models for validation and testing

	**Treatment**	**Primary phenotype or target**	**Biological relevance to glaucoma**
**IOP-elevating agents**	Glucocorticoids<br>- DEX<br>- PA	Increased TM ECM deposition and stiffening	Inducing secondary ocular hypertension and glaucomatous TM phenotype
	TGFβ2	Profibrotic TM ECM remodeling	Increasing outflow resistance in glaucomatous TM
**IOP-lowering agents**	α2-adrenergic agonist<br>- Brimonidine	Reduced aqueous humor and increased uveoscleral outflow	Therapeutic IOP-lowering
	β-adrenergic agonist<br>- Isoproterenol	Influencing aqueous humor dynamics	IOP-lowering in rabbits but no IOP-modulating effect in primates or humans
	Adrenergic agonist<br>- Epinephrine	Reducing aqueous humor production and increasing trabecular outflow	Therapeutic IOP-lowering
	EP2 receptor agonist<br>- Butaprost	Smooth muscle relaxation, cytoprotection, and modulation of ECM remodeling via EP2-mediated cAMP signaling	Experimental IOP-lowering
	latrunculin-B (Lat-B)	Increasing outflow facility and decreasing IOP via disruption of F-actin structure	Experimental IOP-lowering
	Mycotoxin<br>- Cytochalasin B	Increasing trabecular outflow by altering actin-dependent outflow resistance	Experimental IOP-lowering
	Prostaglandin analogs<br>- Latanoprost	Increasing uveoscleral outflow by targeting prostaglandin F (FP) receptor	Therapeutic IOP-lowering
	ROCK inhibitors<br>- Netarsudil<br>- Y27632	Increasing trabecular outflow facility via ROCK-mediated TM relaxation	Therapeutic IOP-lowering<br>Experimental IOP-lowering
	Adenosine A1 receptor agonist<br>- trabodenoson	Increasing MMP-mediated ECM turnover and enhancing conventional outflow facility	Experimental IOP-lowering
	Nitric oxide donor<br>- NCX 667	SC and TM relaxation and increase of outflow facility	Experimental IOP-lowering
	Nitric oxide-donating bimatoprost<br>- NCX 470	SC and TM relaxation and enhanced conventional outflow facility	Late-stage experimental IOP-lowering
**IOP-modulating molecules**	Autotaxin	Increased IOP by elevated autotaxin activity through the LPA signaling	Contributing to ocular hypertension
	Lysophosphatidic acid (LAP)	An IOP-modulating lipid that elevates IOP	Potential IOP-lowering by inhibition of autotaxin–LPA pathway

https://doi.org/10.3390/bioengineering13030291

	Recombinant ANGPTL7 and its blocking antibody	TM ECM remodeling Elevated ANGPTL7 results in increased IOP and TM dysfunction	Anti-ANGPTL7 eliminates the reduction in outflow facility
	TRPV4 agonist - GSK101<br>TRPV4 antagonist - HC-067047	TRPV4— a pressure sensor in the outflow pathway and bidirectional regulator of IOP	GSK101 increases perfusate pressure;<br>HC-067047 reduces outflow facility and perfusate pressure
**Other compounds**	All-trans retinoic acid	Anti-fibrotic ECM remodeling, cytoskeletal organization, and reduction of inflammatory and stress-response signaling	Modulation of key pathways involved in TM dysfunction
	Calcium-chelating agent - Na2EDTA	Breaking calcium-dependent junctions that increases Schlemm's canal permeability	Potential role of calcium sensitive junctions in outflow resistance
	Citicoline	Neuroprotection of RGC	Neuroprotective compound
	Eyedrop preservative - Benzalkonium chloride (BAK)	Toxic and proinflammatory effects on ocular surface and potentially on the TM	Toxicological evaluation of antiglaucoma eyedrops and preservatives
	H_2O_2	Induced oxidative stress	Simulation of glaucomatous stress conditions
	iTRAB®<br>- Polyphenols and fatty acids	Anti-inflammatory and antioxidant effects	Potential to counteract H2O2-induced oxidative stress damage in HTM
	mTOR inhibitors	Influencing TM biology—reducing TGF-β2–induced fibrosis and altering ECM and cytoskeletal pathways that contribute to outflow resistance	Potential relevance to IOP homeostasis
	Riboflavin	Photosensitizer for UV-induced corneal collagen crosslinking	Alteration of biomechanics of collagen for 3D HTM culture
	Verteporfin	Suppression of YAP/TAZ-driven mechanotransduction	Modulation of fibrotic, stiffness-driven changes that contribute to TM dysfunction

https://doi.org/10.3390/bioengineering13030291

Table S2. Representative perfusion studies using HTM or HSC cells grown on filters

Cells	Filter Membrane Inserts / Perfusion	Hydraulic Conductivity (μL/min/mmHg/cm^2)	Reference
HTM	0.45-μm pore size Millicell filters (HATF) / Perfused under an elevated reservoir at a pressure of 5 mmHg	0.3-2.0<br>30-fold increase (10 μM cytochalasin B for 40-50 min)	[182]
HSC	0.45-μm pore size Millicell filters (PIHA01250, mixed cellulose esters (MCE)) / Perfused under an elevated reservoir with a resistor of 0.5 mm Hg/μL/min)	1.0-2.0<br>1.5-3.8 (100 μM isoproterenol for 3-4 h)	[185]
HSC (multiple cell lines)	0.4-μm pore size Snapwell filters / Perfused under an elevated reservoir with an initial pressure at 6 mmHg and decreases gradually	0.08-5.79<br>0.18-10.79 (5 mM Na_2EDTA for 5 min)	[186]
HTM	0.4-μm pore size Millicell filters (#PIHP 03050, polycarbonate) / Perfused under an elevated reservoir at 5 mmHg	0.35-1.31<br>1.04 to 1.30-fold increase (10 minutes post-laser irradiation)	[183]
HTM	Millipore filters / Perfused at 5 mmHg	10.43 ± 1.09<br>13.69 ± 0.83 (10 μM Epinpherin)<br>22.12 ± 0.77 (1 μM DEX for 10 min)<br>2.81 ± 0.30 (1 μM DEX for 5 days)	[184]
Fetal HTM<br><br>Adult HTM	0.45-μm pore size Millicell filters (#PIHA01250, MCE) / Perfused at 20 μL/min using a syringe pump	2.78 ± 1.03<br>1.24 ± 0.72 (0.5 μM DEX for ~10 min)<br>2.41 ± 1.18<br>1.29 ± 0.29 (0.5 μM DEX for ~10 min)	[187]

https://doi.org/10.3390/bioengineering13030291
